# Polysaccharide-mediated modulation of gut microbiota and lysine lactylation: implications for tumor microenvironment remodeling and cancer immunotherapy

**DOI:** 10.3389/fimmu.2026.1924150

**Published:** 2026-10-01

**Authors:** Siwei Yang, Dongmei Long, Weixuan Qiu, Susan L. Morris‐Natschke, Guoxu Ma, Xudong Xu, Zijian Zhao, Yuanxiang Li, Qiongyu Zou, Haifeng Wu

**Affiliations:** 1 Key Laboratory of Research and Utilization of Ethnomedicinal Plant Resources of Hunan Province, Key Laboratory of Hunan Higher Education for Western Hunan Medicinal Plant and Ethnobotany, Hunan Provincial Higher Education Key Laboratory of Intensive Processing Research On Mountain Ecological Food, Key Laboratory of Natural Products Research and Utilization in Wuling Mountain Area, Department of Chemistry & Chemical Engineering, Huaihua University, Huaihua, China; 2 Natural Products Research Laboratories, UNC Eshelman School of Pharmacy, University of North Carolina, Chapel Hill, NC, United States; 3 Beijing Key Laboratory of New Drug Discovery based on Classic Chinese Medicine Prescription, Key Laboratory of Bioactive Substances and Resources Utilization of Chinese Herbal Medicine, Ministry of Education, Institute of Medicinal Plant Development, Chinese Academy of Medical Sciences and Peking Union Medical College, Beijing, China

**Keywords:** gut microbiota, immunotherapy, lactate, lactylation, polysaccharides, tumor microenvironment

## Abstract

Despite the revolutionary clinical benefits of immune checkpoint inhibitors (ICIs), their therapeutic efficacy is largely hampered by the immunosuppressive tumor microenvironment (TME) as well as primary and acquired drug resistance. Natural polysaccharides serve as promising immunomodulators to boost anti-tumor immunity, yet their systematic regulatory network remains poorly characterized. Accumulating evidence demonstrates that gut microbiota and its fermentative metabolites, including short-chain fatty acids and lactate, govern systemic immune surveillance and determine patient responsiveness to ICIs. Meanwhile, lysine lactylation (Kla), a lactate-dependent epigenetic modification, drives glycolytic reprogramming and facilitates tumor immune evasion. This mechanistic review systematically synthesizes current literature and proposes a hypothetical triple regulatory axis: natural polysaccharides act as prebiotics to reshape gut microbial composition and modulate lactate homeostasis, thereby connecting polysaccharide effects, gut microbiota metabolism and lysine lactylation signaling. We comprehensively elaborate how this axis may mediate epigenetic remodeling and immune-cell functional plasticity to reverse TME-derived immunosuppression and confer potential synergistic benefits with anti-PD-1/PD-L1 immunotherapy, benefits so far largely supported by preclinical observations. Notably, although fragmented observational evidence supports this conceptual cascade, direct experimental proof validating the complete causal chain is still lacking. By constructing an integrated metabolic-epigenetic-immune crosstalk framework, this review provides theoretical foundations for developing polysaccharide-based microbiota-targeting adjuvants to overcome ICI resistance. We further summarize major translational bottlenecks and discuss preclinical as well as clinical strategies targeting this cascade to advance tumor immunotherapy.

## Graphical Abstract

**Figure:**
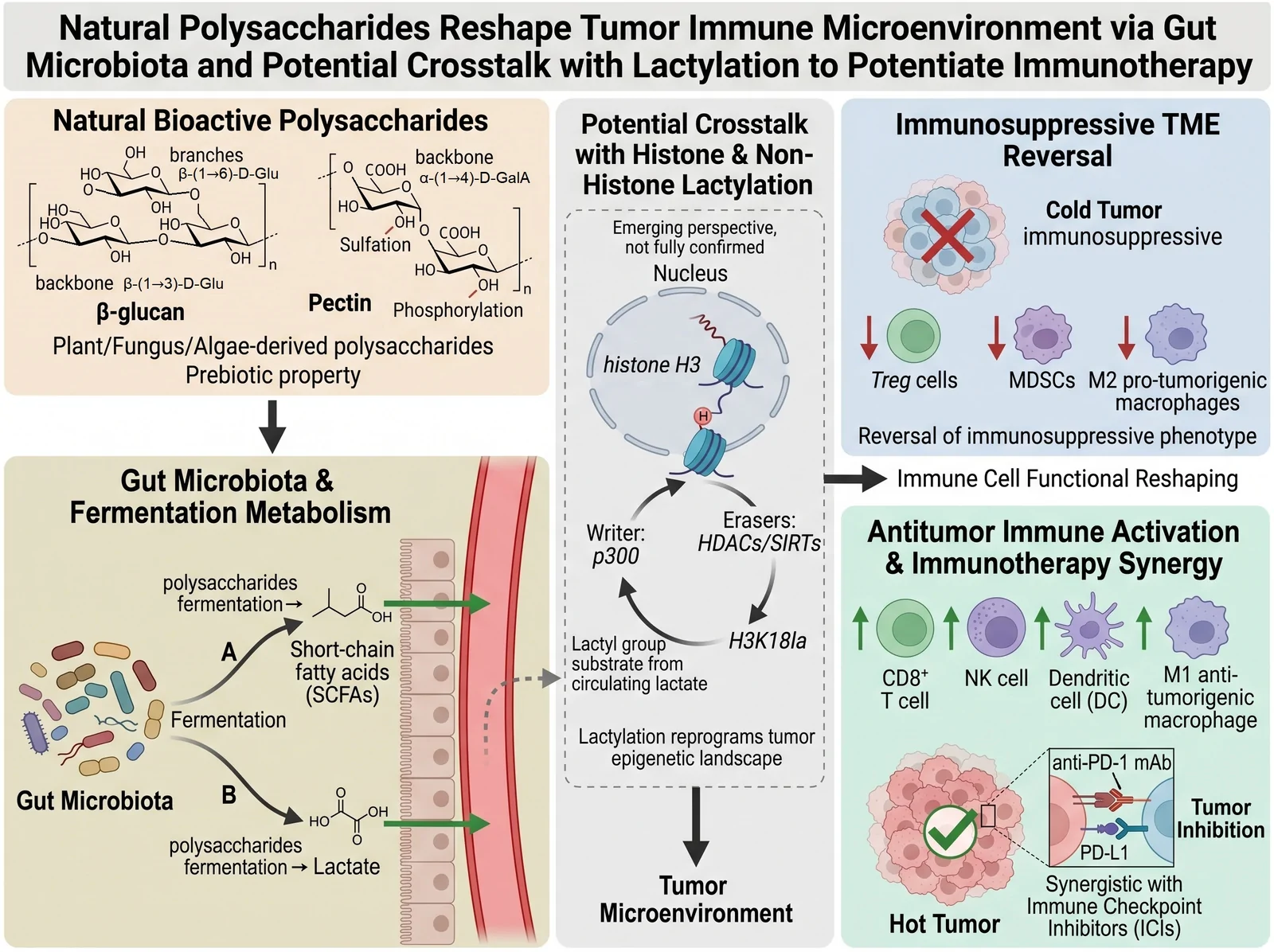
Natural polysaccharides act as prebiotics to remodel gut microbial structure and fermentative metabolism, increasing the generation of short-chain fatty acids (SCFAs) and lactate. Lactate serves as a substrate for histone and non-histone lysine lactylation in the tumor microenvironment (TME). This triple regulatory axis linking polysaccharides, gut microbiota and lactylation rewires immune cell homeostasis, eliminates immunosuppressive TME features, converts immune-cold tumors to immune-hot tumors, and synergistically potentiates anti-tumor immunotherapy efficacy.

## 1 Introduction

Polysaccharides, as natural high-molecular-weight biopolymers, have garnered significant attention due to their diverse biological activities, particularly their immunomodulatory properties. Derived from various natural sources including plants, fungi, algae, and microorganisms, polysaccharides exhibit a broad spectrum of bioactivities such as antioxidant, anti-tumor, antiviral, hypoglycemic, and lipid-lowering effects, as well as the capacity to regulate intestinal flora and immune responses (1–3). Their structural complexity—encompassing monosaccharide composition, molecular weight, glycosidic linkages, branching patterns, and three-dimensional conformations—critically governs their biological functions and therapeutic potential (4). Notably, the correlation between physicochemical modifications (e.g., sulfation, acetylation, and depolymerization) and subsequent alterations in bioactivity has become a pivotal area of investigation, providing a rational basis for the design of novel polysaccharide-based therapeutics (4, 5).

As promising natural bioactive agents, certain natural polysaccharides exert direct antitumor activity, reshape intestinal microbial structure, and mediate the crosstalk between microbial metabolism and host immune homeostasis, forming a vital microbiota-metabolism-immune regulatory axis (6). In particular, bioactive polysaccharides derived from natural sources (e.g., ginseng and *Astragalus*) exert potent immunomodulatory effects primarily through targeting macrophage polarization, thereby modulating cytokine profiles and enhancing systemic anti-tumor immune function (7). Collectively, polysaccharides are regarded as high-value natural immunomodulators with great potential for synergistic cancer immunotherapy.

However, the therapeutic efficacy of cancer immunotherapy is severely limited by the tumor microenvironment (TME), a complex ecosystem characterized by metabolic dysregulation that drives tumor immune evasion. Historically viewed as a metabolic waste product, lactate has been redefined as a critical signaling molecule and substrate for a novel epigenetic modification known as histone lysine lactylation (Kla) (8).

The tumor microenvironment (TME) constitutes a metabolically dysregulated ecosystem that drives immune evasion and compromises cancer immunotherapy efficacy. Long regarded as a metabolic byproduct, lactate is now recognized as a key signaling molecule and substrate for histone lysine lactylation (Kla), an epigenetic modification that rewires gene expression to sustain immunosuppressive TME phenotypes (9, 10). As a pivotal metabolism-linked post-translational modification, lactylation covalently attaches glycolysis-derived lactyl moieties to histone and non-histone lysine residues (10). TME lactate accumulation stemming from the Warburg effect promotes widespread lactylation, thereby modulating gene programs governing tumor progression, immune escape, and therapeutic resistance (11, 12).

Histone lactylation regulates key processes such as DNA repair, metabolic reprogramming, immune cell function, and inflammatory responses within the TME, thereby shaping the dynamic balance of tumor-host interactions (13, 14). Importantly, lactylation affects the polarization of immune cells like macrophages towards immunosuppressive phenotypes and modulates the expression of immune checkpoint molecules, which are pivotal in the efficacy of immunotherapies (10, 15). Notably, marine algal polysaccharides are proposed as potential therapeutic agents targeting the lactate-lactylation axis, although direct experimental evidence remains to be established (9). Therapeutic strategies targeting lactylation-related enzymes or lactate metabolism hold promise for remodeling the TME and enhancing responses to immune checkpoint inhibitors (ICIs), offering new avenues for cancer treatment (9, 10).

Beyond histone lactylation, non-histone protein lactylation also critically shapes the immunosuppressive TME. For instance, lactylation of apolipoprotein C-II (APOC2) at K70 stabilizes the protein, promotes extracellular lipolysis and free fatty acid release, and subsequently induces Treg accumulation and resistance to anti-PD-1 therapy in non-small cell lung cancer (16). Parallel to metabolic–epigenetic reprogramming, the gut microbiota functions as a systemic modulator of immune homeostasis via microbial-derived metabolites—including short-chain fatty acids (SCFAs) and bile acids—that collectively remodel tumor immunotherapy responsiveness (17). As a dynamic microecological community, the gut microbiota orchestrates host immune profiles and metabolic patterns, wherein SCFAs and bile acids are indispensable for systemic immune orchestration and intestinal-metabolic homeostasis (18, 19).

Gut microbiota dysbiosis is closely involved in tumor initiation and development, and can reshape the TME by disrupting immune surveillance and inflammatory signaling networks (20, 21). Accumulating studies have confirmed that gut microbiota and its metabolites markedly affect the efficacy of ICI-based immunotherapy by regulating the infiltration and activation of tumor-infiltrating immune effector cells (22). Microecological regulation strategies, including fecal microbiota transplantation, probiotic and prebiotic supplementation, and dietary intervention, can effectively optimize gut flora structure, boost anti-tumor immunity, and improve therapeutic benefits (20, 23). Moreover, the gut–liver axis fully reflects the sophisticated interaction between microbial metabolites and tumor immunity. In hepatocellular carcinoma, in particular, gut dysbiosis accelerates TME remodeling and mediates chemotherapy and targeted drug resistance (24, 25).

Accumulating evidence demonstrates that bioactive polysaccharides reshape gut microbiota structure and metabolism, thereby modulating host immune homeostasis and tumor-related metabolic pathways (40). Microbial alterations induced by polysaccharides further affect circulating lactate availability, which may potentially modulate lactylation patterns in the TME, forming a potential bridge between gut microbial metabolism and tumor epigenetic reprogramming (8, 17). In this context, certain natural polysaccharides may exert synergistic regulation on gut microecology and lactate-dependent lactylation, which could contribute to remodeling the immunosuppressive TME and improving the efficacy of cancer immunotherapy.

Immunotherapy has revolutionized cancer treatment by harnessing the immune system to target tumor cells. However, its effectiveness is often limited by the immunosuppressive TME and metabolic adaptations of cancer cells (26, 27). The interplay between polysaccharides, gut microbiota, and lactylation provides a potential novel paradigm to address these challenges: polysaccharides, via their immunostimulatory and microbiota-modulating activities, may indirectly affect lactylation-mediated epigenetic reprogramming in the TME by modulating lactate availability, thereby restoring immune surveillance and enhancing responses to ICIs (9, 28). For instance, specific polysaccharides reprogram tumor-associated macrophages toward the M1 phenotype and boost T cell-mediated anti-tumor immunity, concurrently intersecting with lactylation-related signaling pathways (28).

Furthermore, targeting lactylation has been demonstrated to overcome immunotherapy resistance and remodel the immunosuppressive TME in pancreatic ductal adenocarcinoma, highlighting its potential as a key therapeutic node to sensitize tumors to immune checkpoint blockade (29). Lactylation modulation can convert the TME from an immune-cold to an immune-hot state, potentiating lactylation-targeted interventions (30) and complementing polysaccharide-based immunoregulation (9). Thus, integrating polysaccharide-based interventions with microbiota modulation and lactylation-targeted therapies offers a promising strategy to synergistically remodel the TME and improve immunotherapeutic outcomes.

Nevertheless, current evidence derives from isolated studies, and direct experimental validation of the complete polysaccharide–gut microbiota–lactylation regulatory axis remains lacking. This review integrates fragmented evidence regarding polysaccharide-microbiota immunomodulation and further proposes a novel triple regulatory axis linking microbial remodeling and lactylation reprogramming. We also highlight current research gaps and outline prospective experimental directions for future mechanistic verification.

## 2 Polysaccharides, lactylation, and gut microbiota: key players in tumor immunotherapy

The therapeutic potential of natural polysaccharides in oncology has expanded beyond simple immune adjuvants toward modulation of systemic metabolism and gut microbial ecology. Polysaccharides act through three complementary mechanisms: (1) intrinsic structural features mediating direct immune recognition (2) hypothesized capacity to regulate metabolic-epigenetic modifications including lactylation; and (3) prebiotic-driven remodelling of the gut microbiome. This section analyses these three components to build a framework for understanding their combined effects on the tumour immune microenvironment.

### 2.1 Structural characteristics and immunomodulatory functions of polysaccharides

The immunomodulatory potency of natural polysaccharides is intrinsically determined by their complex structural architecture, rather than merely their polymeric nature. Distinct from small-molecule agents, polysaccharides display broad structural diversity originating from monosaccharide composition, glycosidic linkages, branching and three-dimensional conformation. This section describes how structural motifs govern receptor recognition and downstream immune activation.

#### 2.1.1 Chemical structure and classification

Immunomodulatory specificity of polysaccharides arises from hierarchical structural organization spanning primary to tertiary levels. As heterogeneous macromolecules, polysaccharides consist of monosaccharide units connected via stereospecific glycosidic bonds. Such structural diversity enables engagement of pattern-recognition receptors (PRRs) and shapes gut-microbiota interactions.

Polysaccharide architecture can be described across three hierarchical levels:

1. Primary Structure: monosaccharide composition, glycosidic linkage positions (e.g., α-1,4; β-1,3), and chain length.
2. Secondary Structure: ordered backbone conformation, exemplified by the triple-helix geometry of bioactive β-glucans.
3. Tertiary Structure: overall three-dimensional molecular packing in solution (**Figure 1**).

**Figure 1:**
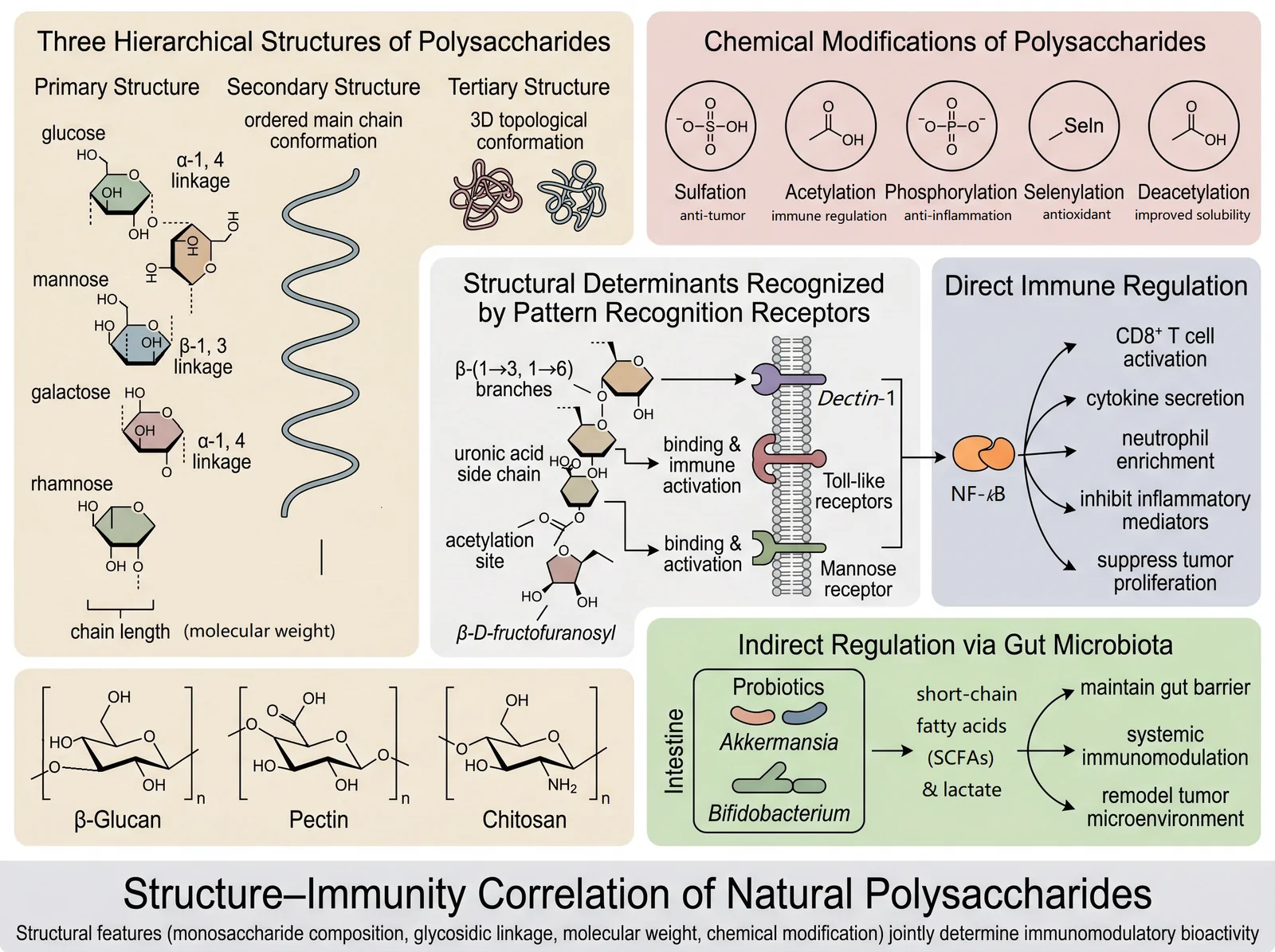
Hierarchical structural features and chemical modifications of natural polysaccharides determine dual immunoregulatory pathways. Primary-to-tertiary structures together with molecular weight represent key structural parameters. Representative examples include β-glucan, pectin and chitosan. Chemical derivatization confers diversified bioactivities. Polysaccharides exert immunomodulation through two routes: direct activation of pattern recognition receptors triggering NF-κB signalling; and indirect reshaping of intestinal commensals to generate SCFAs and lactate, with potential systemic immunomodulation and TME-remodelling consequences.

Representative examples illustrate this spectrum: β-Glucans (derived from yeast, fungi, and cereals) typically feature β-(1→3)-linked backbones with β-(1→6)-branched glucose residues. Their biological activity is strictly contingent upon the maintenance of a triple-helix conformation, which enables high-affinity binding to *Dectin* - 1 and Toll-like receptors to initiate downstream signaling (31, 32). Pectins, rich in galacturonic acid, are structured into homogalacturonan and rhamnogalacturonan regions, exhibiting complex branching with arabinose and galactose side chains that support anticancer activities (33). Chitosan, derived from deacetylated chitin, is a cationic polysaccharide whose amino group content and molecular weight confer unique functions including gene delivery and immune activation (34).

Beyond linkage patterns, the molecular weight (Mw) of polysaccharides spans a broad range—from low kilodaltons to several megadaltons—critically influencing solubility, viscosity, and receptor accessibility. Advanced structural elucidation techniques, including methylation analysis, nuclear magnetic resonance (NMR), and mass spectrometry, have revealed detailed correlations. For instance, polysaccharides isolated from *Polygonatum kingianumare* fructans predominantly composed of β-D-fructofuranosyl and *α*-D-glucopyranosyl linkages; notably, acetylation modifications and mannose residues regulate their immunostimulatory potency through NF-κB signaling activation (35). Similarly, polysaccharides from *Saposhnikoviae Radixexhibit* backbones of galacturonic acid and rhamnose with arabinose and galactose branches, conferring immunomodulatory effects by enhancing neutrophil density and reducing inflammatory mediators (36).

The bioactivity differences among polysaccharides are closely tied to these structural features. β-Glucans with β-(1→3) backbones and β-(1→6) branches and triple-helix conformations exhibit potent immunomodulatory activities, whereas low molecular weight fractions enriched in uronic acids demonstrate enhanced antioxidant properties (32, 37). Furthermore, chemical modifications such as sulfation, acetylation, phosphorylation, and selenylation further diversify functions. These modifications often enhance anti-inflammatory, antitumor, and hypoglycemic activities by altering molecular charge, conformation, and receptor interactions (38, 39). For example, selenized *Artemisia sphaerocephala* polysaccharides exhibit increased antiproliferative effects against tumor cells, attributed to the incorporation of selenite groups at specific positions without degrading the polysaccharide backbone (38).

Ultimately, the interactions between polysaccharides and the immune system are mediated through the recognition of specific structural motifs by PRRs, such as *Dectin* - 1, Toll-like receptors, and mannose receptors on immune cells. The solubility and molecular size of β-glucans influence their ability to activate these receptors, leading to downstream signaling cascades that modulate cytokine production and cellular responses (32). Moreover, specific structures can selectively modulate gut microbiota composition, promoting beneficial bacteria and short-chain fatty acid production, which indirectly influence systemic immunity (28, 40). Thus, the heterogeneity in monosaccharide composition, glycosidic linkages, branching degree, and molecular weight orchestrates the specific immunological outcomes elicited by different polysaccharides.

In summary, natural polysaccharides exhibit remarkable structural diversity, which directly dictates their immunomodulatory potential. Classification by monosaccharide composition, glycosidic linkages, and molecular architecture provides a framework for understanding their distinct bioactivities, while advanced analytical techniques enable precise structural characterization to support structure-function correlations. This knowledge is essential for harnessing polysaccharides as safe and effective immunomodulators, particularly for applications targeting gut microbiota homeostasis and tumor microenvironment remodeling to enhance cancer immunotherapy.

#### 2.1.2 Immunomodulatory effects on innate and adaptive immunity

Building on the aforementioned structure-activity relationships, natural polysaccharides exert broad immunomodulatory activities primarily through the recognition of pattern recognition receptors (PRRs), including Toll-like receptor 4 (*TLR4*) and C-type lectin receptor *Dectin* - 1, which are abundantly expressed on innate immune cells. Chitosan preferentially engages *TLR4*, while β-glucan-type polysaccharides and fucoidan act as primary ligands for *Dectin* - 1; ligation of polysaccharides to these receptors initiates intracellular signaling cascades that bidirectionally remodel the tumor immune microenvironment: they activate antitumor effector immune populations while mitigating immunosuppressive cellular compartments. As illustrated in **Supplementary Figure S1**, this receptor-mediated recognition enhances phagocytosis, antigen presentation, and pro-inflammatory cytokine release, thereby orchestrating robust innate and adaptive antitumor immune responses.

For instance, *Ophiopogon japonicus* polysaccharide liposomes (OPL) target miR-4796 to de-repress the TLR4-NF-κB signaling axis in Kupffer cells. This cascade elevates nitric oxide and iNOS production, boosts phagocytic capacity, and upregulates surface markers CD14 and MHC II to facilitate antigen presentation (41). Similarly, *Tremella fuciformis* polysaccharides (TFP) drive M1 macrophage polarization in B16 melanoma co-culture models. Via MAPK/NF-κB signaling, TFP upregulates M1 markers iNOS and CD80, stimulates secretion of NO, IL-6 and TNF-α, and ultimately triggers melanoma cell apoptosis (42). *In vivo* subcutaneous B16 tumor models further confirm that TFP reshapes tumor transcriptomic profiles, attenuating malignant phenotypes and strengthening systemic anti-tumor immune surveillance (43).

The immunomodulatory effects of polysaccharides are also mediated by their capacity to induce cytokine secretion that reprograms the inflammatory state of the immune microenvironment. For example, *Arctium lappa* L. polysaccharides (ALP) inhibit pro-inflammatory cytokines including IL-8, IL-6, IL-1β, and TNF-α by downregulating the TLR4/NF-κB signaling pathway in macrophages and intestinal epithelial cells, thereby exerting antioxidant and anti-inflammatory effects (44). *Codonopsis pilosula* polysaccharides exhibit structure-dependent immunomodulatory activities, where variations in monosaccharide composition and molecular weight influence the secretion of cytokines such as TNF-α and IL-1β by THP-1 macrophage-like cells, highlighting the importance of polysaccharide structural features in immune regulation (45). *Ephedrae* polysaccharides (PE) demonstrate the ability to rebalance Th1/Th2 and Th17/Treg cell populations, reducing inflammatory cytokines like IL-17A and TNF-α in asthma models, which underscores their role in modulating immune cell subsets and cytokine milieu (46).

At the molecular level, polysaccharides engage PRRs to trigger intracellular signaling pathways including NF-κB, MAPK, and PI3K/Akt, leading to the activation and proliferation of macrophages, dendritic cells, and natural killer cells. This activation enhances the secretion of cytokines such as TNF-α, IL-6, IL-1β, and IFN-γ, which collectively orchestrate the immune response and improve immune surveillance in tumor and inflammatory microenvironments (28, 47). β-Glucans from *Saccharomyces cerevisiae*, for example, reduce bacterial adhesion to intestinal cells and stimulate macrophage phagocytosis and internalization of pathogens, illustrating the dual role of polysaccharides in innate immune activation and microbial defense (48). Moreover, polysaccharides can modulate the balance of T helper cell subsets, particularly restoring the Th1/Th2 equilibrium, primarily achieved through three key molecular pathways: TLR4-MyD88-dependent pathway; Bidirectional regulation of cytokines; JAK/STAT signaling regulation (49).

In addition to direct immune cell activation, polysaccharides influence the immune microenvironment by regulating cytokine profiles that dictate the inflammatory status. Probiotic-derived polysaccharide capsules have been shown to induce differentiation of regulatory T cells and activate dendritic cells via TLRs and C-type lectin receptors, promoting immune tolerance and alleviating allergic diseases (50). Polysaccharides extracted from spores of *Ganoderma lucidum* can trigger T cell-dependent antitumor immunity and improve the therapeutic response to anti-PD-1 blockade in colorectal cancer; this protective effect relies on reshaping gut microbiota, revealing the tripartite crosstalk among polysaccharides, immune compartments and intestinal flora (51).

Furthermore, polysaccharides can be chemically or physically modified to enhance their immunomodulatory efficacy. For instance, subcritical water treatment of ginger polysaccharides alters their chain conformation and molecular weight, resulting in increased macrophage proliferation, phagocytosis, and secretion of NO and pro-inflammatory cytokines such as IL-6, TNF-*α*, and IL-1β, demonstrating a structure-activity relationship critical for immune activation (52). Nanocomposites of polysaccharides with gold nanoparticles have also been synthesized, showing enhanced macrophage activation and antitumor immune responses compared to free polysaccharides, highlighting innovative strategies to potentiate polysaccharide immunomodulation (53).

Polysaccharides modulate immune functions primarily by activating macrophages and dendritic cells via pattern recognition receptors (PRRs), which triggers cytokine secretion to regulate inflammation, enhance host defense and restore immune homeostasis. Their immunomodulatory effects are closely related to structural features and interactions with gut microbiota, indicating great potential as natural immunotherapeutic agents for cancer and inflammatory diseases.

#### 2.1.3 Current status of polysaccharides in tumor immunotherapy

Polysaccharides, natural macromolecules made of monosaccharides, are promising immune adjuvants for tumor vaccines due to their immunomodulatory properties, enhancing antigen presentation and immune activation by promoting dendritic cell (DC) maturation. For example, polysaccharides from *Lonicera japonica* in exosomes improve lymph node targeting and DC activation, resulting in strong CD8^+^ T cell responses and tumor suppression in mice (54). Similarly, *Astragalus* polysaccharides (APS) have been formulated into nanovaccines that serve both as antigen carriers and immune adjuvants, effectively stimulating DC maturation via receptors such as MR and TLR2/4, thereby improving antigen cross-presentation and cytotoxic T lymphocyte activation (55). Mechanistically, polysaccharides activate signaling pathways such as PI3K/Akt, MAPK, and NF-κB in DCs, enhancing antigen uptake and presentation, which is critical for effective tumor immunotherapy (56). Beyond DCs, polysaccharides modulate additional innate immune populations, including macrophages and natural killer cells, to achieve comprehensive immune activation. Their low toxicity, favorable biocompatibility, and versatile chemical modifiability also support their application as vaccine adjuvants. Polysaccharide-based nanoformulations and hydrogel systems further improve structural stability and tumor-targeted delivery (57). Collectively, these properties enable polysaccharides to orchestrate innate and adaptive immune responses, holding great potential for designing next-generation, high-immunogenicity and safe cancer vaccines.

Immune checkpoint inhibitors (ICIs) have revolutionized cancer immunotherapy, yet their efficacy is largely constrained by immunosuppressive TME and tumor heterogeneity. Polysaccharides effectively remodel the TME and potentiate ICI responses by repolarizing tumor-associated macrophages from the pro-tumoral M2 phenotype to the antitumoral M1 phenotype. For example, the homogeneous Coriolus versicolor polysaccharide CVPW-1 drives M2-to-M1 macrophage polarization through the TLR4/Akt/JNK/NF-κB pathway, inhibits macrophage TREM2 expression, and relieves TME immunosuppression, thereby strengthening CD8^+^ T cell-dependent antitumor immunity (58).

*Pholiota adiposa* polysaccharide-coated selenium nanoparticles (PAP-SeNPs) activate the TLR4/MYD88/NF-κB pathway to reverse M2-like TAM polarization, enhancing T cell immunity and improving hepatoma therapy outcomes (59). Similarly, Ganoderma lucidum polysaccharide-based nanocarriers synergize with chemotherapeutic and CD47/SIRPα blockade to re-educate TAMs toward the M1 phenotype, remodel the immunosuppressive TME, and potentiate antitumor immunity (60). Polysaccharides also enhance dendritic cell function and T cell activation, which are critical for effective ICI therapy. Notably, sea cucumber polysaccharides (SCP) were shown to overcome resistance to anti-PD-1 therapy in colorectal cancer models by modulating gut microbiota composition, increasing beneficial bacteria such as *Bifidobacterium*, and augmenting cytotoxic CD8^+^ T cell infiltration in tumors (61). This gut microbiota-mediated mechanism highlights a novel axis through which polysaccharides can enhance immunotherapy. Furthermore, *polyporus* polysaccharide combined with a CD47-targeting peptide delivered via dissolvable microneedles demonstrated synergistic effects by promoting immune checkpoint degradation and repolarizing TAMs, resulting in enhanced antitumor efficacy in melanoma and breast cancer models (62). Moreover, the polysaccharide from *Menispermum dauricum* DC rhizome has been demonstrated to ameliorate chemotherapy-induced injury and reverse the tumor immunosuppressive microenvironment, further supporting the broad potential of polysaccharides in combination cancer therapy (63). Similarly, *Crocus sativus* petal polysaccharides exert potent anti-tumor effects by reconstructing the immunosuppressive tumor microenvironment, activating immune effector cells and inhibiting pro-tumor immune cells (64). Collectively, these studies demonstrate that polysaccharides not only act as immune adjuvants but also remodel the TME and gut microbiota to synergize with ICIs.

Notably, polysaccharide-based nanomedicines have been widely explored as tumor microenvironment-responsive platforms for combination cancer immunotherapy, enabling targeted delivery and controlled release of immunotherapeutic agents to enhance therapeutic efficacy (65, 66). Specifically, biotinylated marine polysaccharide nanoparticles were recently reported to synergistically induce immunogenic cell death and remodel the tumor microenvironment, leading to potent antitumor activity against colorectal cancer (67).

These nanosystems have been developed to co-deliver chemotherapeutics and immunomodulators, achieving amplified antitumor immunity (68). Of note, a multifunctional armored nanoemulsion containing elemene has been developed for colorectal cancer therapy, which integrates ferroptosis induction and gut homeostasis restoration, further highlighting the potential of polysaccharide-based nanoplatforms in combination cancer treatment (69).

The application modes and underlying mechanisms of polysaccharides in tumor immunotherapy are summarized in **Figure 2**.

**Figure 2:**
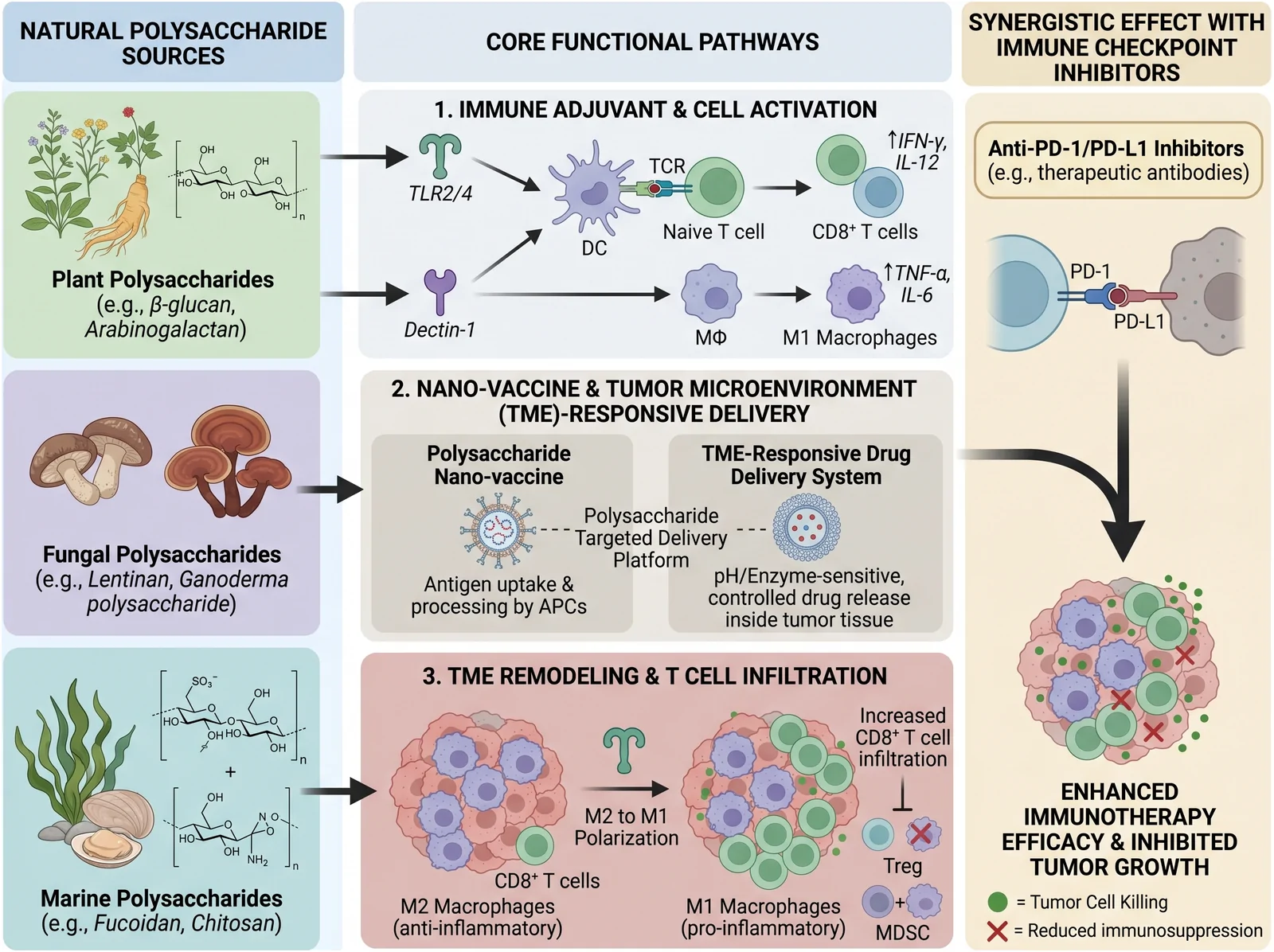
Natural polysaccharides as multi-functional modulators for tumor immunotherapy and synergistic cancer treatment. Polysaccharides derived from plant, fungal, and marine sources enhance anti-tumor immunity through three core mechanisms: innate immune cell activation, tumor microenvironment (TME)-responsive nano-delivery system construction, and TME remodeling via M2-to-M1 macrophage polarization. Furthermore, polysaccharide formulations exert synergistic anti-tumor effects when combined with PD-1/PD-L1 immune checkpoint inhibitors, ultimately boosting immunotherapy efficacy and restraining tumor progression.

### 2.2 Biological functions of lactylation and its role in tumors

#### 2.2.1 Discovery and molecular mechanisms

First reported in 2019, lactylation is a post-translational modification (PTM) that conjugates lactyl groups to protein lysine residues. This finding established a direct mechanistic bridge between cellular metabolism, epigenetic regulation and tumor progression.

Long-considered merely a glycolytic waste product, lactate acts as the substrate for lactylation (70). Distinct from histone acetylation or methylation, lactylation dynamically mirrors intracellular lactate abundance, coupling metabolic flux to transcriptional output.

As summarized in **Figure 3**, glycolysis-derived lactate is imported into cells through monocarboxylate transporters (MCTs) and converted to lactyl-CoA, the obligate substrate for lysine lactylation. It should be emphasized that the full enzymatic machinery governing lysine lactylation remains incompletely resolved. HDAC2 and HDAC3 are well-validated histone lysine delactylases (erasers). By contrast, p300 has been proposed as a candidate lactyltransferase (writer), and SIRT1/2 are suggested as potential delactylases; however, these proposed enzymatic activities remain partially debated and have not been consistently reproduced across different experimental systems. Both histone lactylation (e.g., H3K18la) and non-histone-protein lactylation modulate downstream gene expression to drive tumour metabolic reprogramming and reshape the TME.

**Figure 3:**
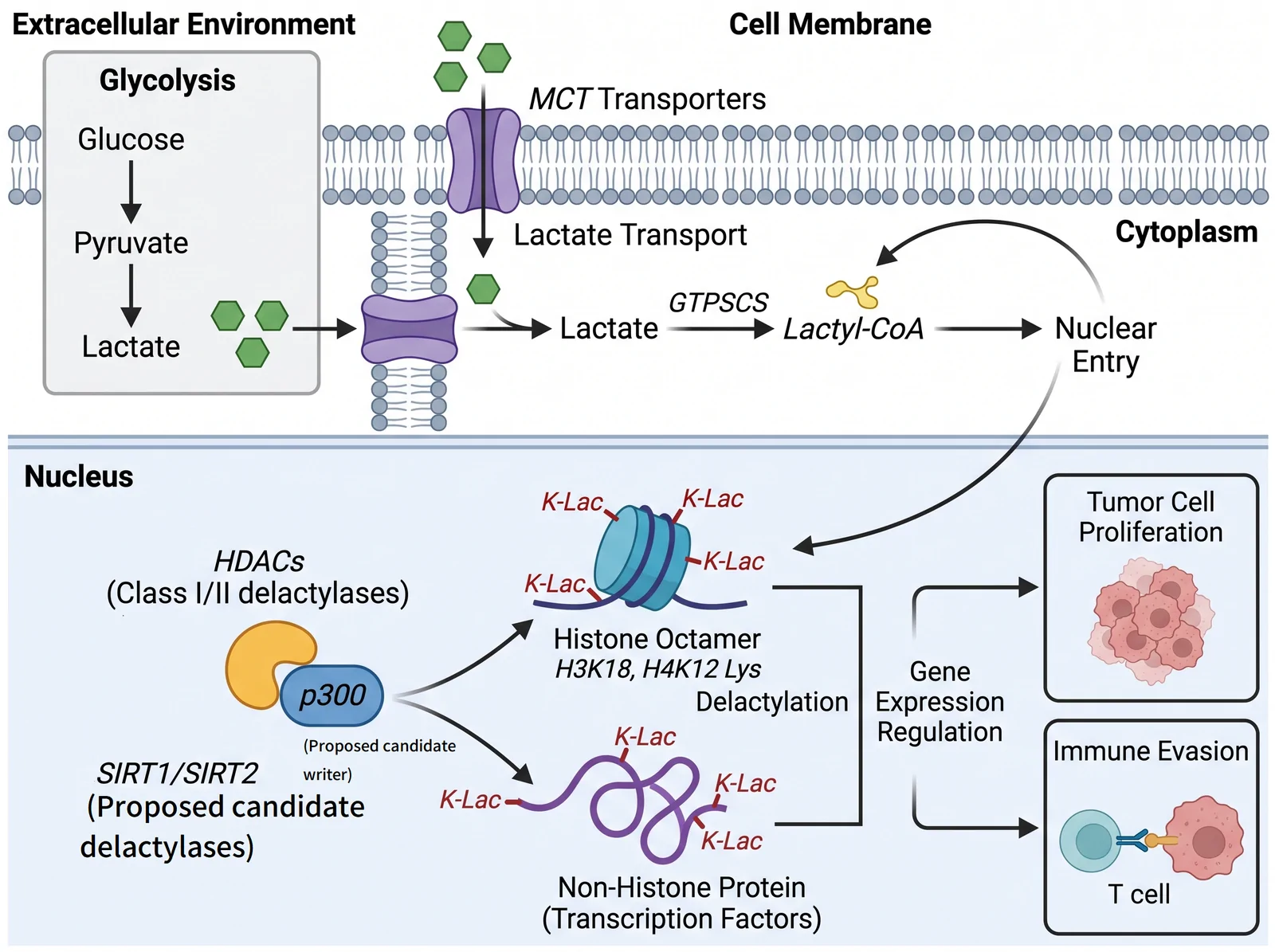
Molecular mechanism of lysine lactylation. Glycolysis-derived lactate enters cells via MCT transporters and is converted to lactyl-CoA, the obligate substrate for lysine lactylation. Multiple candidate regulatory enzymes have been proposed for this reversible modification of histones and non-histone proteins. Well-validated HDAC-family delactylases together with debated candidate enzymes including p300 and SIRTs govern gene programs supporting tumour proliferation, metabolic reprogramming and immune evasion.

Lactylation is a dynamic post-translational modification in which lactate-derived moieties can be transferred to the ϵ-amino group of lysine residues, either enzymatically or non-enzymatically, linking cellular metabolic pathways to epigenetic regulation. As a proposed key candidate writer of this modification, p300 has been reported to catalyze the transfer of lactyl groups to histones at sites including H3K18 and H4K12, yet this activity is not universally accepted (71). Conversely, certain HDAC isoforms function as confirmed delactylases (erasers) to remove these modifications, highlighting the reversible and regulated nature of this process (72). Notably, nuclear lactyl-CoA synthetases like GTPSCS promote the synthesis of lactyl-CoA, a critical cofactor for enzymatic lactylation, thereby influencing the expression of oncogenic genes (73).

Lactate’s role as a metabolic intermediate is central to the lactylation mechanism, serving as a bridge between glycolytic activity and epigenetic control. Elevated lactate levels, often observed under hypoxic conditions or in tumors exhibiting the Warburg effect, enhance lactylation, which in turn modulates transcriptional programs involved in inflammation, immune responses, and cellular differentiation (74). This metabolic-epigenetic nexus allows cells to adapt to environmental and physiological changes by dynamically tuning gene expression through lactylation. Importantly, lactylation is not restricted to histones; non-histone proteins involved in diverse cellular functions including metabolism, DNA repair, and immune regulation are also lactylated, expanding the functional repertoire of this modification (71, 75).

The discovery of lactylation has opened new avenues for understanding how metabolic intermediates regulate epigenetic states and cellular phenotypes. It provides a molecular basis for the crosstalk between metabolism and gene regulation, with implications across a spectrum of physiological and pathological contexts such as cancer progression, immune modulation, neurodegeneration, and aging (76, 77). The dynamic regulation of lactylation by lactate availability and modifying enzymes underscores its potential as a biomarker and therapeutic target. Interventions aimed at modulating lactylation levels or the activity of its regulatory enzymes hold promise for treating diseases characterized by metabolic dysregulation and aberrant epigenetic landscapes (30, 78). Future research is warranted to fully elucidate the complete enzymatic machinery, substrate specificity, and functional consequences of lactylation in various cellular contexts.

In summary, the identification of lactylation as a novel histone and protein modification mediated by lactate represents a paradigm shift in the understanding of metabolic-epigenetic interplay. Lactate’s dual role as a metabolic substrate and epigenetic modifier links cellular metabolic states directly to gene expression and protein function, offering new insights into disease mechanisms and therapeutic strategies.

#### 2.2.2 Regulation of tumor cell metabolism

Lactylation, driven by lactate derived from glycolysis, plays a pivotal role in regulating tumor cell metabolism by enhancing glycolysis and energy production. As a key metabolic-epigenetic nexus, it modifies both metabolic enzymes and histones, directly affecting glycolytic flux through enzymes such as G6PD to promote NADPH production essential for tumor growth (79). This modification facilitates the crosstalk between glycolysis and PPP, thereby supporting anabolic processes and malignant phenotypes in cancer cells. Moreover, lactylation affects the activity of mitochondrial enzymes such as malic enzyme 2 (ME2), where lactylation at lysine 352 enhances enzymatic function and maintains redox homeostasis, promoting tumor proliferation; notably, SIRT3 deficiency leads to excessive ME2 lactylation, further driving tumor growth (80).

Notably, histone lactylation has been identified as a critical epigenetic bridge linking metabolic reprogramming with chromatin-immune crosstalk in triple-negative breast cancer, where elevated lactate-driven lactylation remodels chromatin status and modulates immune-related gene expression to shape the tumor immune microenvironment (81). Furthermore, non-histone lactylation has been shown to directly stabilize the immune checkpoint PD-L1 by inhibiting its ubiquitin-mediated degradation, promoting tumor immune evasion and cell proliferation, which further underscores the multifaceted role of lactylation in shaping the immunosuppressive TME (82). Additionally, lactylation of the small GTPase *Rac2* has been identified as a key regulator of the immunosuppressive microenvironment in lung adenocarcinoma brain metastasis, promoting the recruitment of immunosuppressive cells and driving tumor progression, further demonstrating the broad impact of non-histone lactylation on shaping the TME (83). Notably, pan-cancer multi-omics analyses have systematically characterized lactylation-related genes and confirmed their close associations with tumor metabolic dysregulation, microenvironment remodeling, and malignant progression across diverse cancer types (84). These findings underscore lactylation as a dynamic modulator of metabolic enzyme function, enabling tumor cells to adapt their energy metabolism to microenvironmental stresses.

Beyond enzymatic regulation, lactylation also modulates gene expression programs that govern metabolic pathways. Histone lactylation, particularly at sites such as H3K18, has been shown to activate transcription of genes involved in glycolysis and stemness, thereby enhancing tumor cell survival and proliferation under hypoxic conditions (85, 86). For example, in breast cancer, elevated H3K18 lactylation promotes the expression of the transcription factor PPARD, which in turn activates AKT signaling, facilitating cancer cell survival in anaerobic glycolysis environments (87). This epigenetic regulation illustrates how lactylation integrates metabolic cues with transcriptional control to sustain tumor growth and resistance to metabolic stress. Furthermore, lactylation-driven metabolic reprogramming contributes to therapy resistance by altering tumor cell metabolism and promoting immune evasion within the tumor microenvironment (88, 89).

Histone lactylation has also been reported to regulate the expression of long non-coding RNA LINC00152 in response to bacterial LPS, thereby promoting cancer cell invasion and migration (90). Collectively, these findings position lactylation as a key modulator of tumor cell metabolism and the tumor immune microenvironment. Targeting lactylation-modifying enzymes or lactate dynamics could disrupt metabolic flexibility and enhance treatment efficacy, representing a potential therapeutic node that may synergize with other immunomodulatory strategies, including polysaccharide-based interventions (9, 91) (**Supplementary Figure S2**).

#### 2.2.3 Modulation of immune cell function in the tumor microenvironment

Lactylation, a novel post-translational modification driven by lactate metabolism, critically regulates immune cell function in the tumor microenvironment (TME). By modulating T cell cytotoxicity, macrophage polarization, and immune checkpoint expression, it plays a central role in shaping anti-tumor immunity and driving tumor immune evasion. In CD8^+^ T cells, histone lactylation at specific lysine residues initiates genes for T cell activation, linking metabolism with epigenetic regulation and enhancing cytotoxicity and antitumor immunit (92, 93). Similarly, macrophages, as pivotal components of the TME, undergo lactylation-dependent polarization shifts. Lactylation promotes the transition of macrophages toward an anti-inflammatory, reparative phenotype, which, while beneficial in tissue repair, can paradoxically facilitate tumor immune escape by fostering an immunosuppressive microenvironment. This dual role is mediated through lactylation-driven transcriptional reprogramming that alters cytokine profiles and immune signaling pathways, thereby modulating macrophage functions in gastrointestinal cancers and other malignancies (94). Mechanistically, lactylation modifies both histone and non-histone proteins, influencing gene expression patterns that regulate immune checkpoints such as PD-L1 and B7-H3. For instance, lactylation at H3K18la enhances B7-H3 expression via recruitment of transcription factors and co-activators, leading to diminished CD8^+^ T cell cytotoxicity and tumor immune evasion. Inhibition of glycolysis or lactylation pathways restores T cell function and improves immunotherapeutic efficacy in preclinical models (95, 96). Furthermore, lactylation mediates the formation of an immunosuppressive microenvironment by promoting regulatory T cell activity and suppressing pro-inflammatory immune responses, thereby facilitating tumor progression and resistance to immunotherapy (97, 98).

Notably, recent evidence has extended lactylation-mediated immune regulation to additional cellular compartments. EGR1 lactylation has been shown to trigger tumor cell senescence and establish an immunosuppressive TME by recruiting tumor-associated macrophages and regulatory T cells in intrahepatic cholangiocarcinoma (99).

Collectively, lactylation acts as a central metabolic-epigenetic hub that orchestrates immune cell function, shapes the immunosuppressive TME, and determines therapeutic responses to immunotherapy (100, 101). Moreover, a lactylation-related gene signature has been established to accurately predict prognosis and immunotherapy responsiveness in gastric cancer, further validating lactylation as a key determinant of therapeutic outcomes (102). Notably, the exosome-lactate-lactylation axis serves as a novel metabolic-epigenetic circuit that drives tumor immune evasion via TME remodeling (103). These findings position lactate availability as a key modulator of immune cell function, which may be indirectly influenced by gut microbiota-derived metabolites and host metabolic status, representing a potential point of intersection with polysaccharide-mediated immune regulation. Additionally, hypoxia-driven histone lactylation mediates the functional reprogramming of tumor-infiltrating neutrophils toward an immunosuppressive phenotype, further promoting immune suppression and therapeutic resistance in the TME (104) (**Supplementary Figure S3**).

Of note, lactylation exerts distinct regulatory outputs depending on whether it targets histone or non-histone substrates within immune cells. Histone lactylation mainly remodels chromatin accessibility to reprogram global immune-related transcriptional networks. By contrast, non-histone lactylation predominantly modulates protein stability, enzymatic activity, and signal transduction without directly altering chromatin states. Two representative immune-relevant non-histone substrates are FOXP3 and Arg1. Lactylation of FOXP3 stabilizes its protein abundance and reinforces the immunosuppressive properties of regulatory T cells, whereas Arg1 lactylation in tumour-associated macrophages elevates arginase activity and promotes M2-like pro-tumour polarization, jointly consolidating immune-suppressive TME features (XX, YY). Collectively, histone- and non-histone-directed lactylation cooperatively shape immune cell behaviour and immunotherapy responsiveness.

### 2.3 Gut microbiota-tumor immune crosstalk

#### 2.3.1 Composition and key metabolites of gut microbiota

The gut microbiota is a complex and dynamic ecosystem composed of trillions of microorganisms, primarily bacteria, which play a pivotal role in maintaining host health and homeostasis. The dominant bacterial phyla in the human gut include *Firmicutes, Bacteroidetes, Actinobacteria*, and *Proteobacteria*, with *Firmicutes* and *Bacteroidetes* typically constituting the majority of the gut microbial population (105). Within these phyla, key genera such as *Bacteroides, Faecalibacterium, Lactobacillus*, and *Akkermansia* have been extensively studied for their contributions to gut ecology and host physiology.

The composition of gut microbiota is influenced by numerous factors including diet, age, environment, and disease states, which can lead to variations in microbial diversity and abundance (106, 107). For example, dietary patterns rich in fiber promote the proliferation of beneficial bacteria capable of fermenting complex carbohydrates into short-chain fatty acids (SCFAs), whereas diets high in fat or protein may shift the microbial community towards species associated with metabolic dysregulation (108, 109). These systemic and local effects of gut microbial metabolites are illustrated in **Figure 4**.

**Figure 4:**
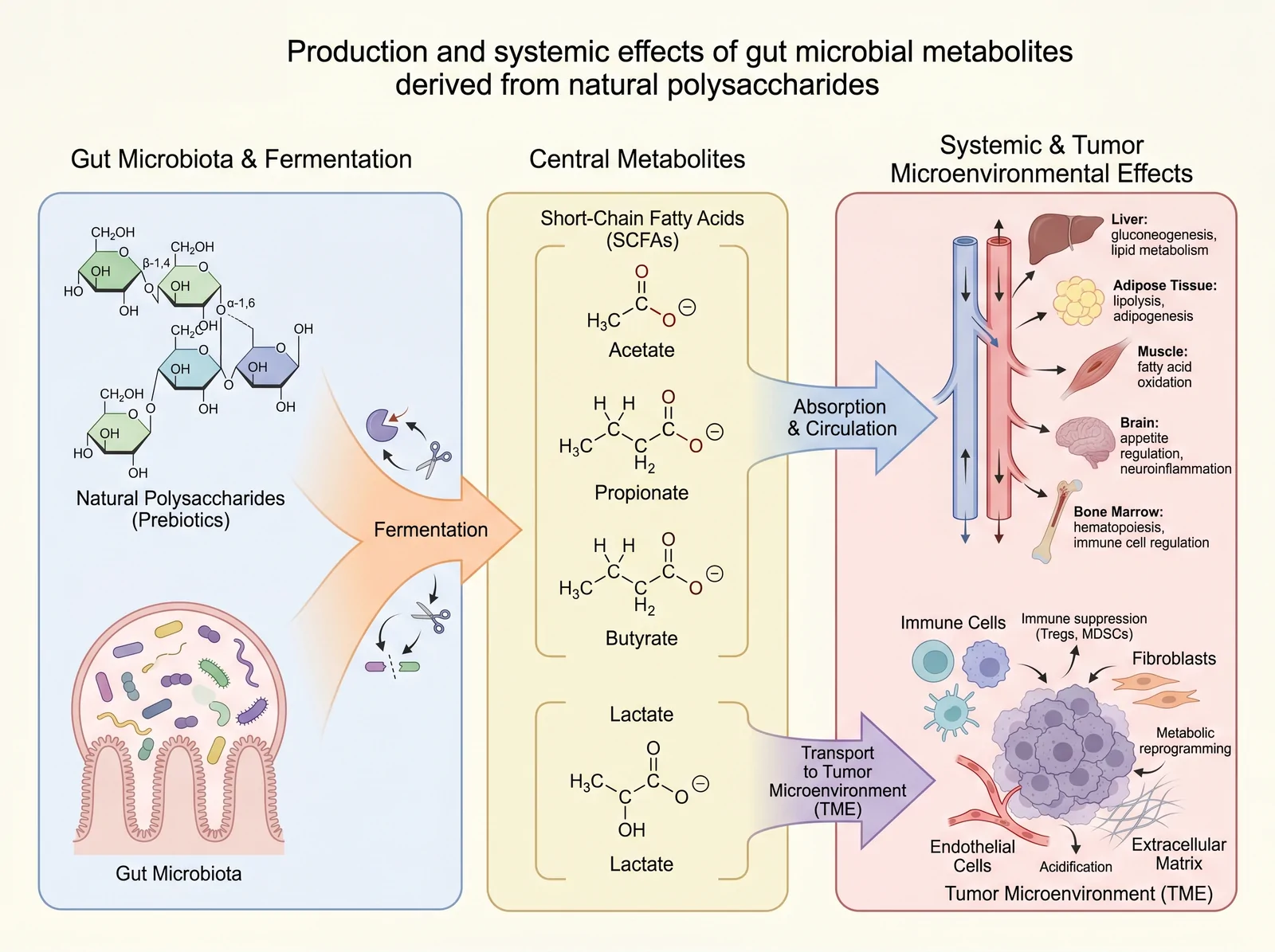
Production and systemic & tumor microenvironmental effects of gut microbial metabolites derived from natural polysaccharides. Natural polysaccharides are fermented by gut microbiota to produce SCFAs and lactate. SCFAs enter systemic circulation to regulate multi-organ metabolism and immune homeostasis, while microbiota-derived lactate translocates into the tumor microenvironment to drive tumor metabolic reprogramming and immunosuppression.

Short-chain fatty acids (SCFAs) such as acetate, propionate, and butyrate are critical metabolic products of the gut microbiota, produced through fermentation of dietary fibers and resistant starches. These SCFAs serve as energy substrates for colonocytes and exert systemic effects by modulating immune responses, maintaining gut barrier integrity, and influencing host metabolism (110). Lactate is another important microbial metabolite generated by lactic acid bacteria including *Lactobacillus* and *Bifidobacterium*; it can be further converted into SCFAs via bacterial cross-feeding to sustain balanced gut metabolic homeostasis. Notably, lactate derived from gut microbiota fermentation is a key link connecting gut dysbiosis to tumor progression, as it can translocate to the TME and regulate lactylation-mediated immune suppression. Other microbial metabolites including bile acid derivatives, tryptophan catabolites and phenolic compounds are also involved in host-microbe interactions, exerting regulatory effects on immune function and multiple physiological processes (111, 112).

The regulatory effects of microbial metabolites on the host immune system are multifaceted. SCFAs, for instance, interact with G-protein coupled receptors (e.g., GPR41, GPR43) on immune cells, promoting the differentiation of regulatory T cells (Tregs) and suppressing pro-inflammatory pathways, thereby maintaining immune homeostasis (113). Butyrate, in particular, inhibits conventional dendritic cell (cDC) development via HDAC inhibition, diminishing their ability to prime T cells and thereby promoting tolerogenic and anti-inflammatory effects in the gut mucosa (114). Tryptophan metabolites produced by gut bacteria, such as indole derivatives, activate the aryl hydrocarbon receptor (*AhR*), which modulates mucosal immunity and barrier function (111). Moreover, microbial bile acid metabolites influence the differentiation and activity of various immune cell subsets, including T helper 17 (Th17), regulatory T (Treg), and innate lymphoid cells (ILCs), further shaping the immune landscape (115). Gut microbial dysbiosis and aberrant metabolite production are closely involved in the development of inflammatory bowel diseases, colorectal cancer, and cancers, thus exerting profound effects on host health (116).

The gut microbiota comprises diverse bacteria that produce bioactive compounds like SCFAs and lactate, which mediate interactions with the immune system, affecting cell differentiation and inflammation. Notably, these microbial metabolites, especially lactate, serve as a key bridge between gut microbiota and the TME, linking microbial dysbiosis to tumor immune evasion. Understanding this composition and its metabolites can inform polysaccharide-based therapeutic strategies to improve immune function, regulate the TME, and treat cancer.

#### 2.3.2 Mechanisms of microbiota influence on tumorigenesis and progression

The gut microbiota significantly influences tumor development and progression, particularly in colorectal cancer, by modulating inflammatory and immune responses to remodel the tumor microenvironment (TME) (117). Gut dysbiosis is closely linked to tumor initiation and progression across various cancer types, including colorectal, hepatobiliary, breast, and pancreatic cancers (118, 119). Mechanistically, gut microbiota regulate tumorigenesis by modulating local and systemic inflammation and by influencing immune cell infiltration and function within the TME. For example, certain microbial species produce metabolites such as short-chain fatty acids (SCFAs), bile acids, and tryptophan derivatives that can either promote anti-tumor immunity or facilitate immune evasion depending on their nature and concentration (120–122). These metabolites affect differentiation and activity of immune cells including T cells, macrophages, natural killer (NK) cells, and dendritic cells, thereby shaping immune surveillance and tumor progression (123, 124).

Gut microbiota can also influence tumor progression by altering metabolic pathways within the TME. For instance, microbial dysbiosis can lead to metabolic imbalances such as increased glutamine metabolism in tumors, which supports cancer cell proliferation and neovascularization, a metabolic reprogramming process tightly mediated by gut microbial composition (125). Furthermore, microbial components and toxins can activate pro-inflammatory signaling pathways, such as NF-κB and Wnt/β-catenin, contributing to tumor-promoting inflammation and epithelial transformation (126). The gut microbiota also modulates the expression of non-coding RNAs, including circular RNAs and microRNAs, which regulate epithelial-mesenchymal transition and cancer stemness, thereby facilitating metastasis (127).

Notably, the regulatory role of gut microbiota in tumorigenesis and progression is closely associated with immune surveillance and tumor immune escape, which are core links connecting microbiota to tumor development. The gut microbiota’s influence extends beyond local tumor sites to systemic immune modulation. The microbiota can regulate immune checkpoints and the efficacy of immunotherapies such as immune checkpoint inhibitors (ICIs) by affecting T cell exhaustion, macrophage polarization, and antigen presentation (128–130). For example, gut microbiota can promote the reprogramming of tumor-associated macrophages from immunosuppressive M2 phenotypes to antigen-presenting M1-like phenotypes, enhancing anti-tumor immunity (123). Additionally, commensal bacteria can stimulate effector T cell responses and modulate cytokine production, thereby influencing tumor immune escape and response to therapy (131, 132). Dysbiosis is also implicated in immune evasion by tumors, as altered microbial communities can impair immune surveillance and promote an immunosuppressive microenvironment (133, 134).

Clinical and preclinical studies have demonstrated that interventions targeting the gut microbiota, such as fecal microbiota transplantation, probiotics, prebiotics, and dietary modifications, can restore microbial balance and improve therapeutic outcomes in cancer patients (20, 135, 136). These strategies can enhance immune responses, reduce adverse effects of immunotherapy, and potentially reverse tumor immune escape mechanisms. Moreover, gut microbiota profiles are emerging as predictive biomarkers for cancer prognosis and immunotherapy responsiveness (130, 137).

In summary, the gut microbiota influences tumorigenesis and tumor progression through a complex interplay involving regulation of inflammation, immune surveillance, metabolic reprogramming, and modulation of the tumor microenvironment. Dysbiosis disrupts these processes, facilitating tumor immune escape and progression, whereas restoration of microbial homeostasis offers promising avenues for enhancing cancer prevention and therapy. Beyond these well-characterized microbial metabolites including SCFAs, bile acids and tryptophan catabolites, lactate-dependent lysine lactylation represents another key signalling node bridging gut microbiota and anti-tumor immunity. Unlike the above-mentioned metabolites that are predominantly microbial products, lactate is of dual origin: it can be generated by gut microbial fermentation as well as intrinsic tumour glycolysis. Functionally, lactate not only acts as a soluble signalling molecule, but also serves as the direct substrate for epigenetic lactylation modification, enabling transcriptional reprogramming in both tumour cells and multiple immune populations. Nevertheless, lactate-mediated lactylation should not be considered as a dominant independent pathway; rather, it constitutes one important component embedded within the broader microbiota-metabolite-immune regulatory network.

##### 2.3.2.1 Microbiota influence on tumor progression via regulation of inflammation and immune surveillance

The gut microbiota critically modulates tumor progression by influencing inflammatory responses and immune surveillance mechanisms. Chronic inflammation triggered by microbial dysbiosis can create a pro-tumorigenic environment through the sustained activation of inflammatory pathways such as NF-κB and STAT3, which promote cellular proliferation, angiogenesis, and DNA damage (126, 138). Specific bacterial species, including *Fusobacterium nucleatum* and enterotoxigenic *Bacteroides fragilis*, have been implicated in promoting inflammation-associated colorectal carcinogenesis by producing virulence factors that disrupt epithelial integrity and activate immune cells (139, 140). These bacteria can also modulate the recruitment and polarization of immune cells within the TME, leading to an immunosuppressive milieu that favors tumor growth.

Conversely, beneficial commensals and their metabolites, such as SCFAs produced by butyrate-producing bacteria like *Eubacterium rectale*, can suppress inflammation and enhance anti-tumor immunity by promoting regulatory T cell differentiation and cytokine production (141, 142). The balance between pro-inflammatory and anti-inflammatory microbial signals is therefore pivotal in determining tumor fate. Gut microbiota also regulate innate immune cells such as NK cells, which mediate tumor cell cytotoxicity. Microbiota depletion or dysbiosis impairs NK cell infiltration and function, facilitating tumor immune evasion and progression, as demonstrated in pancreatic cancer models (119).

Moreover, the gut microbiota influences adaptive immune responses by shaping T cell repertoires and activation states. Microbial antigens and metabolites can modulate T helper cell differentiation, cytotoxic T lymphocyte activity, and immune checkpoint expression, thereby affecting tumor immunosurveillance and responsiveness to immunotherapy (124). For example, gut microbiota-derived indole-3-acetic acid has been shown to induce immunosuppressive IL-35 expression, ameliorating precancerous inflammation and inhibiting tumorigenesis (122).

In clinical settings, modulation of gut microbiota composition has been associated with improved outcomes in immune checkpoint blockade therapies. Specific bacterial taxa correlate with enhanced CD8^+^ T cell infiltration and reduced T cell exhaustion within tumors, highlighting the microbiota’s role in potentiating immunotherapy efficacy (128, 137). Conversely, dysbiosis can contribute to immune checkpoint inhibitor resistance and immune-related adverse events, such as colitis, by disrupting immune homeostasis (143).

Collectively, these findings underscore the gut microbiota’s dual role in regulating inflammation and immune surveillance, which critically influences tumor progression. Therapeutic strategies aiming to restore or manipulate gut microbial communities hold promise for enhancing anti-tumor immunity and overcoming immune evasion.

##### 2.3.2.2 Relationship between gut microbiota dysbiosis and tumor immune escape

Gut microbiota dysbiosis is intricately linked to tumor immune escape, a hallmark of cancer that allows malignant cells to evade immune detection and destruction. Dysbiotic microbial communities can promote immunosuppressive TME remodeling by altering the recruitment, differentiation, and function of immune cells. For instance, dysbiosis often leads to increased abundance of bacteria that induce regulatory T cells (Tregs) and myeloid-derived suppressor cells (MDSCs), which suppress effector T cell activity and facilitate tumor tolerance (123, 134).

Mechanistically, microbial metabolites such as secondary bile acids and tryptophan derivatives modulate immune checkpoints and cytokine secretion, contributing to immune evasion. Dysregulated bile acid metabolism by gut microbiota can activate receptors like *FXR* and *TGR5*, leading to the expansion of immunosuppressive macrophages and inhibition of cytotoxic T cell responses (121). Similarly, altered tryptophan metabolism affects aryl hydrocarbon receptor (*AhR*) signaling, which influences immune cell ferroptosis and tumor-promoting inflammation (132, 144).

Furthermore, dysbiosis can impair antigen presentation by dendritic cells and reduce the efficacy of immune checkpoint inhibitors by promoting exhausted T cell phenotypes and suppressive macrophage subsets, such as Spp1^+^ tumor-associated macrophages (128, 145). The microbiota also modulates the expression of non-coding RNAs that regulate immune evasion pathways, enhancing tumor metastasis and resistance to therapy (127).

Clinical studies have demonstrated that correcting dysbiosis via fecal microbiota transplantation or probiotic administration can restore anti-tumor immunity, reduce immune escape, and improve responses to immunotherapy (135, 136). These interventions increase beneficial microbial taxa that produce immunostimulatory metabolites, promote effector T cell infiltration, and reprogram suppressive immune cells within the TME (**Supplementary Figure S4**).

#### 2.3.3 Regulation of immunotherapy responses by gut microbiota

Gut-microbiota diversity and composition are critical modulators of anti-PD-1/PD-L1 immune checkpoint inhibitor (ICI) efficacy in cancer treatment (146–148). Multiple clinical and pre-clinical observations link higher gut microbial α-diversity to favourable ICI responses across melanoma, non-small-cell lung cancer (NSCLC), colorectal cancer (CRC), and hepatocellular carcinoma (HCC) (149).

Nevertheless, microbial taxonomic biomarkers for ICI responders exhibit poor reproducibility across human cohorts. Three landmark melanoma studies (146–148) reported partially conflicting bacterial signatures: Gopalakrishnan et al. identified enrichment of *Faecalibacterium* and *Ruminococcaceae*; Matson et al. described distinct beneficial commensals; and Routy et al. highlighted *Akkermansia*. Even within Gopalakrishnan’s two melanoma cohorts, only Ruminococcaceae showed partial cross-cohort consistency. Such irreproducibility partly accounts for variable clinical outcomes of ICI-refractory tumour FMT trials.

In NSCLC, positive associations have been reported: PD-1-blockade responders display higher microbial richness, alongside elevated *Faecalibacterium* and *Akkermansia*, taxa correlated with systemic IL-12 and IFN-γ and enhanced T-cell priming (150, 151). However, numerous null and non-replicated results also exist, indicating gut-microbiota-derived predictive biomarkers are not yet ready for routine clinical application.

Mechanistically, gut microbiota shapes antitumour immunity by regulating both innate and adaptive compartments within the TME. Microbial metabolites including short-chain fatty acids (SCFAs), tryptophan derivatives and bile acids boost activation and infiltration of cytotoxic CD8^+^ T cells, dendritic cells and NK cells to drive antitumour defence (152–154). Hexa-acylated lipopolysaccharides (LPS) from specific gut bacteria augment anti-PD-1 efficacy via TLR4-dependent immune activation, whereas other LPS isoforms exert inhibitory effects (155). These findings reflect the complexity of microbial regulation, where both community diversity and taxon-specific functions jointly determine immunotherapy outcomes.

Microbial profiles also influence immune-related adverse events (irAEs); certain taxa predispose patients to colitis and other immunotherapy-associated toxicities (157, 158). Collectively, gut microbiota composition and diversity hold promise as predictive biomarkers for ICI response and toxicity, demanding further prospective validation and mechanistic investigation (156, 157).

Microbiota-targeted interventions represent attractive strategies to boost cancer immunotherapy. Fecal microbiota transplantation (FMT) and probiotic supplementation yield encouraging pre-clinical and early-phase clinical results. Preclinically, FMT from ICI responders transfers heightened antitumour immunity to germ-free or antibiotic-treated mice, restoring beneficial microbes and their metabolites to improve ICI responses (152, 154). For instance, supplementation with Bifidobacterium infantis or Akkermansia muciniphila promotes intratumoural CD8^+^ T-cell recruitment and activation and potentiates anti-PD-1 therapy (151, 159).

Clinical evidence, although still emerging, supports the feasibility and potential efficacy of microbiota modulation in cancer patients. Small-scale clinical trials have reported that FMT from ICI responders to non-responders can induce clinical responses and favorable immunological changes in melanoma patients refractory to immunotherapy (154, 160). Similarly, probiotic supplementation with *Lactobacillus* and *Bifidobacterium* species has been investigated as adjuncts to immunotherapy, showing potential to improve treatment outcomes and reduce immune-related toxicities, although results are variable and require larger controlled studies (161, 162). Dietary interventions aimed at promoting beneficial gut microbes through prebiotics or specific nutrients like tryptophan have also been explored. While clinical observations are limited, preclinical evidence indicates that diet-induced modulation of the microbiota can enhance immunotherapy efficacy by increasing SCFA production and T-cell function (163, 164).

Moreover, mechanistic studies reveal that microbiota-based interventions can influence key immune pathways, including the *CXCL10-CXCR3* axis for T cell recruitment and *TLR4* signaling for innate immune activation, thereby reshaping the tumor immune microenvironment to favor antitumor responses (155, 159). However, challenges remain in standardizing microbiota-based therapies, addressing inter-patient variability, and ensuring safety, especially concerning the risk of infections or dysbiosis-related complications. Future directions emphasize the development of precision microbiota therapeutics, including engineered bacterial strains and defined microbial consortia, to optimize immunotherapy outcomes (135, 165) (**Supplementary Figure S5**).

In summary, the modulation of gut microbiota through FMT, probiotics, and dietary strategies represents a promising adjunct to cancer immunotherapy. Ongoing clinical trials and mechanistic research are critical to establish effective, safe, and personalized microbiota-based interventions that can overcome resistance and enhance the durability of immunotherapeutic responses.

## 3 Polysaccharides as regulators of lactylation and gut microbiota for enhanced immunotherapy

Certain natural polysaccharides are biocompatible natural macromolecules capable of reversing the immunosuppressive TME by modulating metabolism and epigenetics, with lysine lactylation as a core target. As introduced in Chapter 2, lactate-dependent lactylation mediates the Warburg effect, tumor metabolic reprogramming and immune evasion. This part illustrates how these carbohydrate molecules regulate lactate pools and lactylation modification to reshape the TME and boost immunotherapy efficacy. Notably, such bioactivities are strongly contingent on polysaccharide backbone architecture, molecular weight, and experimental model systems and cannot be generalized to all carbohydrate polymers.

### 3.1 Polysaccharides remodel the tumor microenvironment via regulating lactylation

Notably, polysaccharide-mediated lactylation regulation exhibits strong cell-type- and context-dependent specificity, reconciling the seemingly contradictory pro- and anti-tumor effects reported in current studies. In general, moderate lactylation in cytotoxic CD8+ T cells strengthens anti-tumor immune function, whereas excessive lactylation in tumor cells, Tregs and MDSCs exacerbates immunosuppressive TME formation. The final regulatory outcome is comprehensively determined by polysaccharide structural features, tumor types, and local metabolic states.

#### 3.1.1 Modulation of lactate metabolism and lactylation levels

Certain natural polysaccharides significantly impact lactate metabolism and protein lactylation by modulating glycolytic pathways in tumor and immune cells, which rely heavily on aerobic glycolysis (the Warburg effect) for lactate overproduction. These agents regulate key enzymes and transporters governing glucose uptake and lactate generation, thereby shifting intra- and extracellular lactate concentrations. In addition, selected polysaccharides foster the proliferation and metabolic activity of lactic-acid-producing bacteria within gut microbiota (166); microbially-derived lactate may enter systemic lactate pools and further shape host metabolic processes linked to lactylation.

In the tumor microenvironment, elevated lactate concentrations serve as substrates for lysine lactylation, a reversible post-translational modification where lactyl groups derived from lactate covalently bind to lysine residues on histones and non-histone proteins. This lactylation process links metabolic reprogramming to epigenetic regulation, influencing gene expression patterns that promote tumor progression, immune evasion, and resistance to therapies (167, 168). Certain natural polysaccharides may modulate lactylation indirectly by altering lactate availability through their effects on glycolysis and lactate metabolism. For example, polysaccharides from tucum fruits demonstrated antitumor effects by modulating oxidative stress and inflammation without impairing hepatic gluconeogenesis from lactate, indicating a nuanced influence on systemic lactate metabolism (169). Moreover, some types of polysaccharides can influence immune cell metabolism and function, potentially affecting lactate production and utilization within the tumor microenvironment.

At the cellular level, certain natural polysaccharides can regulate key glycolytic enzymes and lactate transporters. For instance, the bacterial pathogen *Francisella tularensis* exploits host lactate metabolism by upregulating monocarboxylate transporter 4 (*MCT4*) and lactate dehydrogenase B (*LDHB*) in macrophages, enhancing lactate oxidation to support infection and immune evasion (170). Some polysaccharide members may counteract such mechanisms by modulating these metabolic pathways. Additionally, in bacterial biofilms, lactate production and lactylation regulate biofilm formation and metabolic activity, with polysaccharide metabolism playing a critical role (171). These insights underscore the interplay between polysaccharide metabolism, lactate dynamics, and lactylation.

Furthermore, certain natural polysaccharides influence the gut microbiota composition and metabolic outputs, including short-chain fatty acids and lactate, which can affect host systemic metabolism and immune responses (172). By promoting beneficial microbes such as *Lactobacillus* and *Bifidobacterium* that produce lactate, some types of polysaccharides indirectly regulate lactate pools and downstream lactylation processes. The modulation of microbial metabolites by polysaccharides also impacts inflammatory pathways and tumor microenvironment remodeling (**Supplementary Figure S6**).

In summary, certain natural polysaccharides modulate lactate metabolism and lactylation via multiple routes: tuning glycolytic flux in tumour-immune cells, shaping lactate-metabolizing gut microbiota, and regulating lactate transporters together with lactylation-related enzymes. Collectively, these events remodel the TME and boost antitumour immunity, supporting the therapeutic prospect of polysaccharides in cancer immunotherapy.

#### 3.1.2 Effects on tumor-associated immune cell function

Certain natural polysaccharides reshape tumour-associated immune-cell function by modulating lysine lactylation, which connects Warburg-effect-driven glycolytic reprogramming to epigenetic transcriptional control within the TME. Glycolysis- derived lactate serves as the substrate for histone lactylation, which determines the differentiation, effector function and antitumour competence of CD8^+^ T cells and dendritic cells (DCs).

In cytotoxic CD8^+^ T lymphocytes, lactate accumulation triggers site-specific histone lactylation at H3K18 and H3K9. Such lactylation marks activate effector gene programs to maintain T-cell tumoricidal capacity; impaired lactylation markedly compromises CD8^+^-T-cell-dependent antitumour immunity (92). Marine algal polysaccharides can correct aberrant T-cell lactylation profiles, recover cytotoxic effector-gene expression, relieve T-cell exhaustion and improve immunotherapy responses.

Dendritic cells are another central effector of lactate–lactylation signaling. Loss of mitochondrial pyruvate carrier (MPC) function in colorectal cancer triggers massive lactate buildup and global hyperlactylation in DCs. Excessive lactylation represses the DC maturation marker CD33, impairs antigen presentation, and suppresses downstream CD8^+^ T cell responses, thereby driving tumor immune escape and resistance to anti-PD-1 therapy (173). Certain natural polysaccharides treatment stabilizes MPC activity to limit lactate overproduction, alleviate pathological histone lactylation in DCs, and rescue DC maturation and cross-priming capacity (174). Consistent with this, oat β-glucan enriches *Faecalibacterium prausnitzii* to produce butyrate and indole-3-propionic acid, which activate DCs via the HDAC8/H3K27ac/NF-κB axis and synergize with anti-PD-1 therapy in colorectal cancer (174).

Tumor-associated macrophages (TAMs), the most abundant immunosuppressive population in the TME, are also profoundly governed by lactate-dependent lactylation. Lactate-derived histone lactylation transcriptionally upregulates repair and immunosuppressive gene programs, pushing TAMs toward a protumor M2 phenotype (175, 176). Some of these polysaccharide candidates can reverse this polarization shift by lowering intratumoral lactate and subsequent lactylation in TAMs. For example, *Lachnum*-derived polysaccharides inhibit S180 sarcoma growth by redirecting TAMs toward an antitumor M1 phenotype, accompanied by reduced histone lactylation and recovered proinflammatory cytokine secretion (177). Consistently, pectin polysaccharides extracted from *Phyllanthus emblica* also drive M1 polarization of TAMs, relieve the “cold” immunosuppressive TME, and exert robust antitumor activity (178).

High lactate concentrations in the TME promote protein lactylation within these cells to amplify their inhibitory potency. In MDSCs, lactylation stabilizes arginase-1 (Arg1), boosting enzymatic activity to deplete local arginine and block T cell activation (179). In Tregs, lactylation of the master transcription factor FOXP3 at lysine 277 enhances protein stability and reinforces Treg immunosuppression (180). As summarized in recent systematic reviews, diverse polysaccharides counteract lactylation-mediated immune suppression by normalizing intratumoral lactate pools—reducing Arg1 lactylation in MDSCs (179) and FOXP3 lactylation in Tregs (180)—simultaneously boosting endogenous anti-tumor immunity and sensitizing tumors to immune checkpoint blockade (9, 180) (**Supplementary Figure S7**).

Although the specific enzymatic targets through which polysaccharides modulate lactylation remain incompletely characterized, accumulating evidence indicates their capacity to remodel immune epigenetic states and cellular plasticity within tumors. Existing research indicates that certain natural polysaccharides may interfere with core lactylation-modifying enzymes such as the lactyltransferase p300, thereby indirectly reshaping the global lactylation landscape in immune cells. This pathway offers an innovative mechanistic explanation for their adjuvant efficacy in tumor immunotherapy (176).

#### 3.1.3 Potential for enhancing immunotherapy efficacy

As illustrated in **Figure 5**, certain natural polysaccharides form a multi-layered regulatory network that profoundly remodels the tumor microenvironment (TME) by targeting the lysine lactylation pathway, acting through both gut microbiota-dependent indirect mechanisms and tumor cell-intrinsic direct mechanisms.

**Figure 5:**
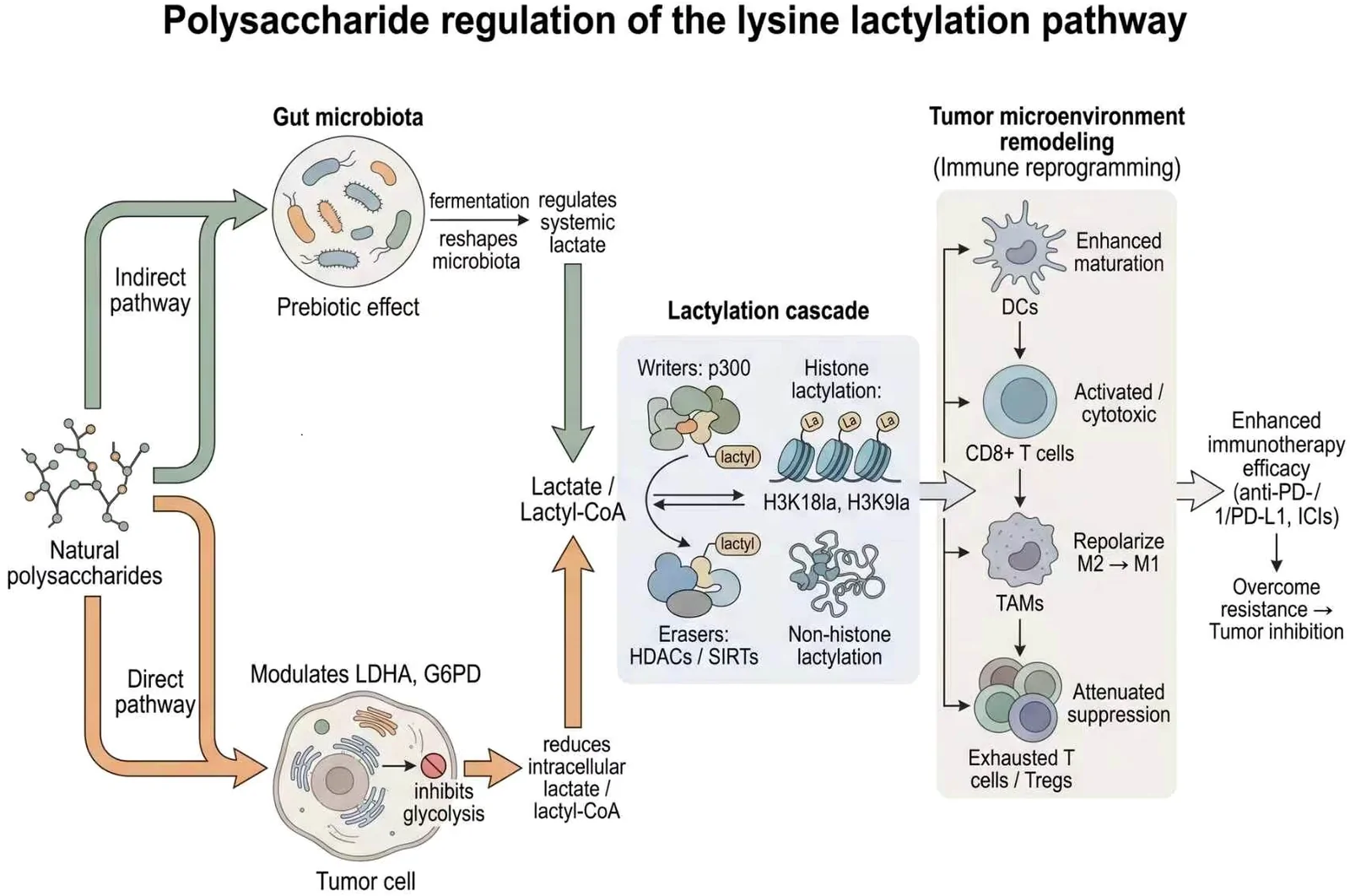
Some natural-polysaccharide-mediated regulation of the lysine lactylation pathway. Certain natural polysaccharides modulate lysine lactylation via dual mechanisms: indirectly, as prebiotics reshaping gut microbiota to regulate systemic lactate availability; directly, by modulating metabolic enzymes (LDHA, G6PD) and glycolysis to alter intracellular lactate/lactyl-CoA levels. These changes drive histone and non-histone lactylation, mediated by p300 and HDACs/SIRTs, ultimately mediating epigenetic and immune regulation in the TME.

Building on the mechanistic insights outlined above, the crosstalk between polysaccharides and lysine lactylation opens promising avenues to boost cancer immunotherapy, particularly the therapeutic response to immune checkpoint inhibitors (ICIs). Cumulative experimental evidence indicates that certain natural polysaccharides remodel lactylation landscapes within the tumor microenvironment (TME), linking tumor metabolic reprogramming to comprehensive immune regulation. Excessive lactate accumulation in tumor tissue drives widespread histone lactylation, which upregulates multiple immune checkpoint molecules and shapes an immunosuppressive cellular landscape featuring M2-type tumor-associated macrophages and exhausted CD8^+^ T cells, ultimately facilitating tumor immune escape (175, 176). Certain Polysaccharides derived from marine algae, medicinal herbs and fungi exert potent antitumor activities by targeting such lactylation-dependent immune inhibitory pathways. Specifically, marine algal saccharides interrupt lactylation-mediated signal transduction, restrain M2 macrophage polarization, restore dendritic cell (DC) maturation and antigen presentation, and collectively reinforce endogenous antitumor immune surveillance (9).

In addition, polysaccharide-based nanomedicines address prominent drawbacks of conventional ICIs including poor stability, insufficient tumor-targeting capacity and uncontrolled *in vivo* release (67, 177). When equipped with lactylation-modulating moieties, these delivery platforms may generate synergistic immune-stimulatory effects. Multiple preclinical models have validated that certain natural polysaccharides can reverse lactylation-induced immunosuppression to sensitize tumors to ICI treatment. For instance, *Codium fragile*-derived polysaccharides amplify anti-PD-L1 therapeutic efficacy by activating DCs and facilitating intratumoral infiltration of cytotoxic T lymphocytes (179). Similarly, *Astragalus* polysaccharide (APS) alleviates lactylation-mediated immunotherapy resistance and optimizes the immunosuppressive TME to strengthen checkpoint blockade efficacy (180). Consistently, direct pharmacological suppression of histone lactylation has been proven to improve immunotherapeutic outcomes in gastrointestinal malignancies, further establishing lactylation as a druggable target for combination tumor therapy (181). Collectively, these observations highlight the great translational potential of polysaccharides as lactylation-regulating immunoadjuvants to recover impaired tumor immune surveillance.

From a translational therapeutic perspective, lactylation represents an innovative actionable target for cancer immunotherapy. The identification of core lactylation-modifying enzymes (e.g., p300 lactyltransferase and deacetylase SIRT2), together with the clarified regulatory roles of lactylation in checkpoint signaling and TME remodeling, lay a theoretical foundation for developing polysaccharide-centered targeted intervention strategies (15, 182, 183). Chemical structural modification of polysaccharides may endow them with potential to suppress pathological lactylation; meanwhile, polysaccharide-based platforms can enable specific delivery of lactylation inhibitors to tumor lesions to overcome immunotherapy resistance (184). Moreover, engineered polysaccharide derivatives serve as efficient vaccine adjuvants that trigger TLR4 signaling, promote DC maturation and antigen-specific T cell responses, and thereby counteract lactylation-driven immune suppression (185).

Notably, H3K9 lactylation in malignant cells has been directly shown to drive CD8^+^ T cell dysfunction and attenuate the efficacy of immunotherapy (186), further validating lactylation as a pivotal target for polysaccharide-mediated intervention. The combination of polysaccharide-based nanocarriers with lactylation-targeting agents presents a promising approach to modify the immunosuppressive TME, enhance immune cell infiltration, and boost ICI therapies, though challenges remain in understanding the molecular mechanisms of polysaccharides in lactylation and translating these insights into clinical applications. Critically, lactylation also acts as a key driver of drug resistance in the tumor microenvironment, making polysaccharide-based interventions targeting this pathway a promising strategy to counteract both immune suppression and therapy resistance (187).

Future studies should identify polysaccharide structures that effectively modulate lactylation, optimize delivery systems, and conduct clinical trials to confirm their efficacy and safety, highlighting the potential of the polysaccharide-lactylation pathway as a novel immunotherapeutic strategy to combat tumor immune evasion and improve cancer treatment outcomes, particularly in gastrointestinal malignancies (15).

### 3.2 Polysaccharides modulate gut microbiota to regulate tumor immunity

Mounting evidence suggests that the anti-tumor activity of selected natural polysaccharides is partially mediated through gut-microbiota remodelling. Acting as prebiotics, these compounds reshape microbial communities, stimulate immunomodulatory metabolites and drive host immune responses that remodel the TME. This section discusses their impacts on microbiota composition, metabolite generation, and microbiota-driven anti-tumor immunity.

#### 3.2.1 Polysaccharides as prebiotics and modulators of gut microbiota composition

Polysaccharides and gut microbiota engage in complex bidirectional crosstalk. On the one hand, gut bacteria can modify polysaccharide structures and thereby alter bioactivity. For example, *Lactobacillus* fermentation remodels Eucheuma spinosum polysaccharide, changing its molecular weight, monosaccharide profile and immunomodulatory potency (188).

Conversely, natural polysaccharides function as prebiotics to shape gut-microbiota composition and metabolism. They selectively foster beneficial commensals to sustain community homeostasis, an effect linked to colorectal-cancer suppression (189). Polysaccharides derived from plants, fungi and algae commonly enrich probiotic genera including *Bifidobacterium* and *Lactobacillus*, as well as SCFA-producing bacteria, while inhibiting pathogenic taxa. Their fermentation efficiency, prebiotic potency and downstream immunometabolic outputs are strongly governed by polysaccharide structure-activity relationships (190). Consistently, natural polysaccharides broadly sustain intestinal homeostasis and gut microbial balance, supporting their reliable prebiotic properties in cancer-modulating contexts (191). As a well-characterized medicinal polysaccharide, *Dendrobium officinale* polysaccharide exerts prebiotic-related therapeutic benefits by reshaping gut microbiota structure, enhancing SCFA production and maintaining intestinal barrier integrity (192).

Notably, seaweed-derived polysaccharides also act as potent prebiotics that modulate gut microbiota composition and promote the generation of SCFAs, further expanding the source diversity of polysaccharides with prebiotic and immunomodulatory activities. Moreover, edible fungal polysaccharides effectively regulate gut microbiota composition and function, thereby promoting host metabolic balance and immune health through microbiota-dependent pathways (193). Likewise, Morchella esculenta polysaccharide attenuates obesity and inflammation while modulating gut microbiota composition, further supporting the prebiotic and systemic regulatory roles of edible fungal polysaccharides (194).

The fermentation of polysaccharides by gut microbiota directly shapes the metabolic output, most notably increasing the production of beneficial short-chain fatty acids (SCFAs) like acetate, propionate, and butyrate. These SCFAs are crucial for gut barrier integrity and immune regulation. Beyond SCFAs, certain natural polysaccharides modulate other key metabolite classes. They influence bile acid metabolism by affecting bacterial transformations, which is vital for lipid metabolism (195). Furthermore, polysaccharides can impact tryptophan metabolism pathways; for example, asparagus polysaccharide was found to reverse aberrant levels of the tryptophan derivative L-glutaminyl-L-tryptophan, linking gut microbiota modulation to systemic metabolic effects (196). This broad regulation of the metabolome underpins the systemic health benefits of polysaccharides.

Collectively, emerging metabolomic and microbial studies have revealed the intricate interactions between polysaccharides and gut microbiota, where microbial fermentation of polysaccharides shapes metabolic profiles and microbial community structure to influence host health (197).

For instance, polysaccharide fractions from *Polygonatum kingianum* (PKP) have been shown to be resistant to digestion and effectively fermented by gut microbiota, particularly promoting *Bifidobacterium* proliferation, indicating strong prebiotic potential (198). Similarly, polysaccharides extracted from *Oudemansiella raphanipes* (OrPs) significantly altered gut microbiota composition in mice, increasing fecal short-chain fatty acids (SCFAs) and beneficial bacterial groups such as *Lachnospiraceae* and *Bifidobacterium*, which are known SCFA producers (199).

Likewise, *Cyclocarya paliurus* polysaccharides optimize gut microbial metabolic function by modulating SCFA synthesis and community structure, further validating the prebiotic and metabolic-regulating activities of natural polysaccharides (200). In addition, *Asparagus officinalis* polysaccharides alleviate atherosclerosis through reshaping gut microbiota and metabolite profiles, highlighting the vital role of polysaccharides in maintaining intestinal homeostasis and systemic metabolism (201).

Furthermore, polysaccharides isolated from *Fritillaria* spp. possess unique structural fingerprints and exert strong gut microbiota-modulating effects, balancing intestinal flora to sustain gut homeostasis (202). Acacia gum polysaccharide also effectively regulates gut microbial assembly to exert favorable prebiotic functions and stabilize intestinal microecology (203).

Moreover, *Ophiopogon japonicus* polysaccharides relieve pulmonary fibrosis by mediating crosstalk between gut microbiota and microbial metabolites, demonstrating that natural polysaccharides can exert systemic regulatory effects through the gut-lung axis (204). In addition, a novel polysaccharide purified from *Astragalus complanatus* seeds displays distinctive structural features and anti-hepatic fibrosis activity, expanding the broad therapeutic prospects of natural polysaccharides in regulating tissue homeostasis and metabolic health (205).

Together, these observations underscore the critical crosstalk between gut microbiota-derived metabolites and the tumor microenvironment, which serves as a key axis linking polysaccharide-mediated microbiota modulation to downstream anti-tumor immune and metabolic effects (206). Furthermore, gut microbiota-derived metabolites play pivotal roles in shaping the tumor immune microenvironment by regulating immune cell infiltration, activation, and effector functions, further linking microbial metabolism to anti-tumor immunity (207). Fermented *Astragalus* polysaccharides (FAPS) also demonstrated the ability to increase beneficial bacteria like *Lactobacillus* and *Enterococcus faecalis* while inhibiting harmful species, thereby enhancing SCFA production and contributing to intestinal homeostasis (208). These findings underscore the role of certain natural polysaccharides in fostering a balanced gut microbial ecosystem by selectively nourishing beneficial microbes. The key mechanisms by which polysaccharides modulate gut microbiota composition and promote SCFA production are illustrated in **Figure 6**.

**Figure 6:**
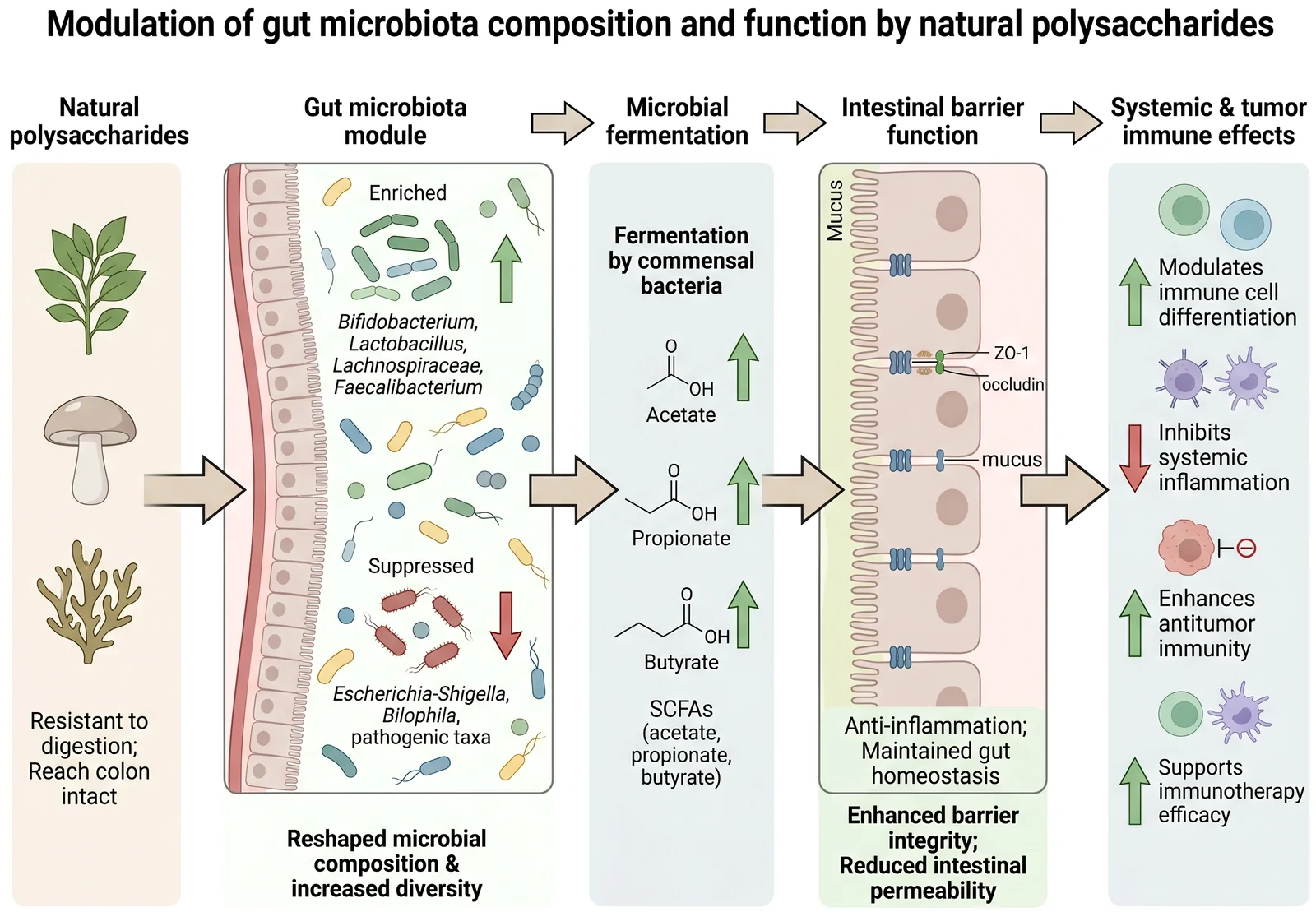
Modulation of gut-microbiota composition and function mediated by certain natural polysaccharides. Certain natural polysaccharides resist host digestion and reach the colon intact, acting as prebiotics to enrich beneficial bacteria and suppress pathogens. These changes promote microbial fermentation and SCFA production, enhancing intestinal barrier integrity and gut homeostasis. Ultimately, these effects modulate systemic immunity, inhibit inflammation, enhance anti-tumor immunity, and support immunotherapy efficacy.

The structural properties of polysaccharides, including molecular weight, monosaccharide composition, and glycosidic linkages, critically influence their prebiotic efficacy. For example, degraded *Acanthopanax senticosus* polysaccharides with reduced molecular weight showed enhanced probiotic effects by promoting colonization of beneficial bacteria such as *Faecalibacterium* and *Paraprevotella*, which contribute to SCFA production and gut barrier protection (209). Similarly, sulfated modification of *Poria cocos* polysaccharides improved their solubility and prebiotic effects, leading to increased abundance of *Streptococcus*, *Bifidobacterium*, and *Lactococcus*, along with elevated SCFA levels and reduced pro-inflammatory cytokines IL-6 and TNF-α (210). These microbial shifts demonstrate that polysaccharide structural characteristics are decisive in remodeling gut microbiota and mediating immunoregulatory responses.

Fermentation of polysaccharides by intestinal commensals generates SCFAs (acetate, propionate, butyrate), which are central to sustaining intestinal barrier homeostasis, regulating mucosal immune reactions, and supplying energy for colonocytes. Multiple studies indicate that supplementation with certain natural polysaccharides increases intestinal SCFA levels, correlating with improved gut barrier function and alleviated inflammation. For instance, *in vitro* fermentation of *Auricularia auricula* polysaccharides (AAP) boosts SCFA production and reshapes gut microbial composition: it raises the Bacteroidetes/Firmicutes ratio and inhibits opportunistic pathogens *Escherichia-Shigella* (211). Consistently, oral administration of Stellariae Radix polysaccharides stimulates the proliferation of *Lactobacillus acidophilus* and *Bifidobacterium longum*, elevating overall microbial diversity and gut health in mice (212). These microbial metabolites not only strengthen the gut barrier but also influence systemic immunity by regulating cytokine production and immune cell differentiation.

Moreover, polysaccharides preserve gut barrier integrity by upregulating tight junction proteins and lowering intestinal permeability. Synthetic prebiotic oligosaccharides such as galactooligosaccharides mitigate inflammation-triggered barrier damage via inhibiting NF-κB signaling and reducing pro-inflammatory cytokine release in intestinal epithelial cell models (213). Strengthened gut barriers block the translocation of pathogenic bacteria and endotoxins, thereby alleviating systemic low-grade inflammation and boosting host immune surveillance.

Most existing research focuses on polysaccharides regulating tumor immunity, while *Astragalus membranaceus*-derived astragalus polysaccharide extends the regulatory spectrum of polysaccharide–microbiota crosstalk to the gut–lung axis and metabolic homeostasis, supporting the broad immunomodulatory potential of natural polys beyond cancer (214).

In summary, certain natural polysaccharides act as efficient prebiotics: they selectively enrich beneficial gut microbes, stimulate SCFA biosynthesis, and reinforce intestinal barriers, jointly mediating immune regulation and gut microecological stability. Their variable structural features and fermentability by gut microbiota underpin their biological functions, rendering them promising nutritional candidates to improve intestinal health and synergistically enhance tumor immunotherapy efficacy. Further research on polysaccharide structure–activity relationships and their metabolic interactions with gut microbiota will clarify underlying molecular mechanisms and advance their clinical and nutritional applications.

#### 3.2.2 Regulation of gut microbial metabolite production

Plant- and fungus-derived polysaccharides serve as vital fermentable substrates for intestinal microbiota, yielding bioactive metabolites including short-chain fatty acids (SCFAs) and lactate to mediate host metabolism and immune function. Accumulating evidence indicates certain representatives among these molecules boost SCFA synthesis by enriching probiotic genera *Bifidobacterium* and *Lactobacillus*.

For instance, longan pulp polysaccharides raised colonic SCFA concentrations by 51.63% and upregulated colonic G-protein-coupled receptor 41 (GPR41), thereby governing whole-body energy balance and mucosal immune signaling (215). Likewise, dietary oat β-(1→3, 1→4)-D-glucan elevated butyrate output and the relative abundance of Lachnospiraceae, a butyrate-producing bacterial family; this microbial shift restored colonic barrier integrity and relieved colon tissue damage triggered by food allergy (216).

SCFAs act as critical signaling mediators to govern immune cell differentiation, pro/anti-inflammatory cytokine secretion, and intestinal epithelial barrier function, collectively conferring potent anti-inflammatory and immunoregulatory effects. Lactate further stabilizes gut microecology by nourishing butyrate-generating commensals. Polysaccharides extracted from *Poria cocos* and sea cucumber both enrich SCFA-producing microbes and elevate luminal lactate concentrations, supporting their prebiotic capacity (217, 218).

It should be emphasized that intratumoral lactate primarily originates from tumour cell-intrinsic glycolysis, which dominates the local lactate pool in the TME. Although gut microbiota-derived lactate can enter systemic circulation, its actual contribution to intratumoral lactate levels under physiological conditions remains unquantified. Direct *in vivo* evidence verifying that gut-derived lactate reaches tumours at functionally effective concentrations is still absent, leaving a critical gap for future stable-isotope tracing validation. Importantly, lactate accumulated following polysaccharide intervention not only maintains intestinal homeostasis but also functions as an essential precursor for lysine lactylation, an epigenetic post-translational modification that rewires metabolic and transcriptional programs within tumor cells.

In particular, increased lactate production provides a critical substrate for protein lactylation, linking polysaccharide-induced microbial metabolism to epigenetic regulation in the tumor microenvironment. The regulatory effects of polysaccharides on the lactylation pathway are illustrated in **Supplementary Figure S8**.

The metabolites derived from intestinal microbiota, especially SCFAs and lactate, remodel signaling within the tumor microenvironment (TME) by binding to *GPCRs* expressed on immune cells. These metabolites exert anti-inflammatory effects, reinforce intestinal epithelial barrier integrity, and modulate gene transcription to suppress inflammatory responses and facilitate regulatory T cell differentiation (219).

Intervention with certain natural polysaccharides reshapes gut microbial composition and their metabolic output, further altering core amino acid metabolic pathways including phenylalanine, tyrosine and tryptophan biosynthesis—pathways tightly linked to local immune regulation in the TME. Bergamot polysaccharides elevated L-phenylalanine and SCFA concentrations, accompanied by attenuated colonic inflammation and upregulated tight junction proteins, indicative of restored mucosal barrier function and immune homeostasis (50).

Moreover, certain natural polysaccharides modulate bile acid metabolism through gut commensals, thereby interfering with farnesoid X receptor (*FXR*) cascades that govern tumor progression and immune balance (220). Cross-talk between microbial metabolites and host receptors (*GPCRs, HDACs, FXRs*, aryl hydrocarbon receptors [*AHRs*]) coordinates immune cell recruitment, cytokine secretion profiles, and intracellular metabolic reprogramming in the TME, ultimately governing tumor proliferation and the therapeutic efficacy of immunotherapy (219, 221).

Additionally, certain natural polysaccharides restore balanced gut microbiota and metabolite pools to relieve chronic systemic inflammation and oxidative stress, two key drivers of a pro-tumorigenic microenvironment. For instance, *Cistanche deserticola* polysaccharides remodel gut flora and boost metabolites such as *D*-proline and histidine, alleviating neuroinflammation and providing new clues for targeting inflammation-associated tumor development (222).

Two representative regulatory axes uncovered in recent research further illustrate this mechanism. On one hand, polysaccharides extracted from *Schizophyllum commune* selectively enrich the beneficial commensal *Faecalibaculum rodentium* and stimulate the production of its metabolite indole-3-lactic acid, mediating broad systemic immunometabolic regulation; this axis serves as a promising target for remodeling the tumor immune microenvironment (223).On the other hand, the human gut commensal bacterium *Bacteroides xylanisolvens* XB1A can specifically recognize and deconstruct xylan from *Dendrobium officinale* via its discrete polysaccharide utilization loci (PULs), converting the macromolecular polysaccharide into small molecules and producing beneficial metabolites such as short-chain fatty acids and folate, which further supports the prebiotic mechanism of this medicinal polysaccharide (224).

In summary, metabolite accumulation triggered by selected polysaccharide candidates activates multiple intracellular signaling pathways to remodel the TME: it enhances tumor immune surveillance, alleviates inflammatory burden and strengthens intestinal barrier function, consequently amplifying the therapeutic potency of cancer immunotherapy.

#### 3.2.3 Evidence for polysaccharide-mediated microbiota-immune crosstalk

Recent evidence shows that certain natural polysaccharides can modulate gut microbiota to enhance immunotherapy efficacy by reshaping the tumor immune microenvironment (TME). Animal studies indicate that polysaccharides (e.g., *Astragalus* polysaccharides, APS) inhibit melanoma tumor growth by altering gut microbiota, reducing immunosuppressive myeloid-derived suppressor cells (MDSCs), and improving CD8^+^ T cell activity. However, combining APS with microbiota-disrupting antibiotics negates these effects, highlighting the gut microbiota’s crucial role in APS’s antitumor activity (225). Similarly, sea cucumber (*Stichopus japonicus*) polysaccharides synergized with anti-PD-1 immunotherapy in MC38 tumor-bearing mice by modulating gut microbiota−increasing beneficial bacteria (e.g., *Akkermansia, Bifidobacterium*) and elevating metabolites like indole-3-carboxylic acid. Fecal microbiota transplantation (FMT) experiments further confirmed that gut microbiota partially mediated this synergy, enhancing cytotoxic CD8^+^ T cell infiltration and reducing intratumoral regulatory T cells (226).

In addition, a selenium-modified polysaccharide from *Acanthopanax senticosus* enhances anti-tumor immunity by reshaping the gut microbiota, increasing SCFA-producing bacteria and butyrate levels; its anti-tumor effect, dependent on the gut microbiota, is verified by microbiota depletion and FMT experiments (227). Similarly, *Ganoderma lucidum* polysaccharides regulate gut microbiota composition and enhance host immune regulation via the gut–liver and gut–brain axes, supporting their prebiotic and immune regulatory functions (228). Notably, *Ganoderma lucidum* polysaccharide-decorated extracellular vesicles enable synergistic antitumor immunotherapy, serving as an advanced biomaterial-based strategy to boost the immune efficacy of polysaccharides (229).

Fucoidan, a marine-derived polysaccharide, also exhibited gut microbiota- dependent antitumor effects by promoting the growth of immunomodulatory bacteria and modulating tryptophan metabolism, which led to increased CD8^+^ T cell proportions and decreased IDO1 expression in tumor tissues (230). These animal model studies collectively highlight that certain natural polysaccharides can act as prebiotics to selectively enrich beneficial microbial taxa, which in turn influence systemic and local immune responses within the TME.

Mechanistically, certain natural polysaccharides modulate immune cell infiltration and function via gut microbiota-dependent cascades. Polysaccharide-driven microbial remodeling generally boosts the production of immunoregulatory short-chain fatty acids (SCFAs), including butyrate, propionate and acetate. For example, *Ganoderma* polysaccharides increase SCFA-producing microbes and modulate Toll-like receptor (TLR) signaling, promoting immune cell activation and triggering tumor cell apoptosis (231).

In cyclophosphamide-immunosuppressed mice, polysaccharides from *Gastrodia elata* restored gut microbial diversity and increased SCFA levels, which correlated with improved intestinal barrier integrity and elevated secretion of immune-related cytokines and immunoglobulins, suggesting a gut microbiota-dependent restoration of immune competence (232). In addition, polysaccharides from *Codonopsis pilosula* enrich intestinal *Lactobacillus*. These microbes drive T cell-mediated anti-tumor immunity and suppress tumor-associated inflammation by regulating neutrophil degranulation and oxidative stress signaling (233). Collectively, these emerging preclinical observations suggest that certain natural polysaccharides remodel gut microbiota composition and metabolite profiles, which may potentiate effector T cell function and constrain immunosuppressive populations, including MDSCs and regulatory T cells. Nevertheless, most current conclusions remain correlative, and definitive causal validation through microbiota depletion, germ-free models, or FMT verification is still lacking (**Supplementary Figure S9**).

Furthermore, *Polygonatum cyrtonema* Hua polysaccharides relieve muscle atrophy and excessive fat lipolysis by restoring gut microecological homeostasis in chemotherapy-induced cancer cachexia models (234).

Translational and preliminary clinical data further suggest that polysaccharide- mediated gut microbiota remodeling improves immunotherapy efficacy. Tumor microenvironment heterogeneity largely restricts the therapeutic response of immune checkpoint inhibitors (ICIs); certain natural polysaccharides can reverse this limitation by reshaping gut microbiota and metabolome, converting the immunosuppressive TME into an immunostimulatory niche (221).

As a typical example, edible plant polysaccharides exert anti-tumor and immunoregulatory effects through the gut microbiota–immune axis, though relevant human *in vivo* evidence remains scarce and requires further validation (235). Emerging evidence suggests selected polysaccharide candidates act as natural adjuvants, leveraging gut microbiota to enhance intratumoral immune cell activity and improve immunotherapy responses. However, challenges remain in identifying effective polysaccharide structures and microbial taxa; future research combining multi-omics, microbiota transplantation, and clinical trials is critical to translate these findings into cancer immunotherapy strategies.

### 3.3 Synergistic mechanisms of polysaccharide-regulated lactylation and gut microbiota

Gut microbiota and protein lactylation cooperatively regulate the TME, with lactate serving as the core metabolic and epigenetic mediator linking both pathways. Natural polysaccharides are hypothesized to remodel intestinal microbial composition and fermentation, thereby altering systemic and local lactate pools to modulate protein lactylation and drive epigenetic reprogramming in tumour and immune cells. This section elaborates lactate as the central bridge connecting microbial metabolism and lactylation, describes the polysaccharide–gut microbiota–lactylation hypothetical axis, and discusses how this crosstalk reverses immunotherapy resistance.

Notably, lactylation functions in a context-dependent manner determined by cell type, tissue location and local metabolite abundance. Moderate lactylation sustains the cytotoxicity and antitumour activity of effector CD8^+^ T cells, whereas aberrant lactylation in tumour cells, Tregs and MDSCs fosters immunosuppression. Similarly, SCFA-mediated HDAC inhibition exerts cell-specific effects: it elevates lactylation in effector CD8^+^ T cells to preserve antitumour function, whereas elevated lactylation driven by HDAC inhibition within suppressive immune subsets can promote immune evasion. Furthermore, polysaccharides differentially remodel lactate and lactyl-CoA levels in systemic circulation versus the tumour niche. Therefore, the final immunological outcome relies on cellular context, tissue compartment and metabolite thresholds, rather than simple upregulation or downregulation of global lactylation. Cumulative primary and correlative experimental findings supporting the above‑mentioned complex regulatory patterns are compiled in **Supplementary Table S1**.

#### 3.3.1 Lactate as the central metabolic bridge: proposed polysaccharide-gut microbiota-lactylation hypothetical axis

Certain natural polysaccharides, gut microbiota and protein lactylation form a hypothesized regulatory axis capable of remodelling the TME. Specific polysaccharides reshape gut microbial structure and fermentative metabolism to elevate lactate production. As a pivotal metabolic intermediate, lactate connects microbial activity to histone lactylation, reprogramming transcriptional profiles in tumour and immune cells and ultimately remodelling the TME. This regulatory paradigm highlights the immunomodulatory function of polysaccharides and provides new insights for TME-targeted tumour therapy (168, 207, 236).

Lactate produced by gut microbial anaerobic fermentation is a pleiotropic signalling metabolite that bridges commensal metabolism and host physiology. As the obligate substrate for lactylation, lactate fuels this post-translational epigenetic modification, which modulates chromatin accessibility and gene transcription. Lactate thus acts both as a microbial fermentation by-product and an epigenetic switch, coupling gut microbial homeostasis to host anti-tumour immunity and malignant progression. Dissecting lactate-dependent signalling illuminates how microbial metabolic shifts remodel cellular phenotypes via lactylation. Together with other epigenetic marks, lactate shapes global epigenetic profiles and regulates gene transcription, contributing to multiple pathologies including cancer and metabolic syndromes (237).

Collectively, current evidence suggests that certain natural polysaccharides enrich beneficial gut microbes and reroute microbial fermentation toward lactate production, which may in turn drive histone lactylation within the TME. This hypothesized sequential cascade could alter immune-cell differentiation and cytokine secretion, reshape tumour-cell epigenetic programming, and establish an immunostimulatory niche favouring tumour immune surveillance and therapeutic responsiveness. As prebiotic agents, these polysaccharides may orchestrate TME epigenetic and immune remodelling, offering plausible yet experimentally unvalidated avenues to improve cancer immunotherapy clinical outcomes (21, 168, 238) (**Figure 7**).

**Figure 7:**
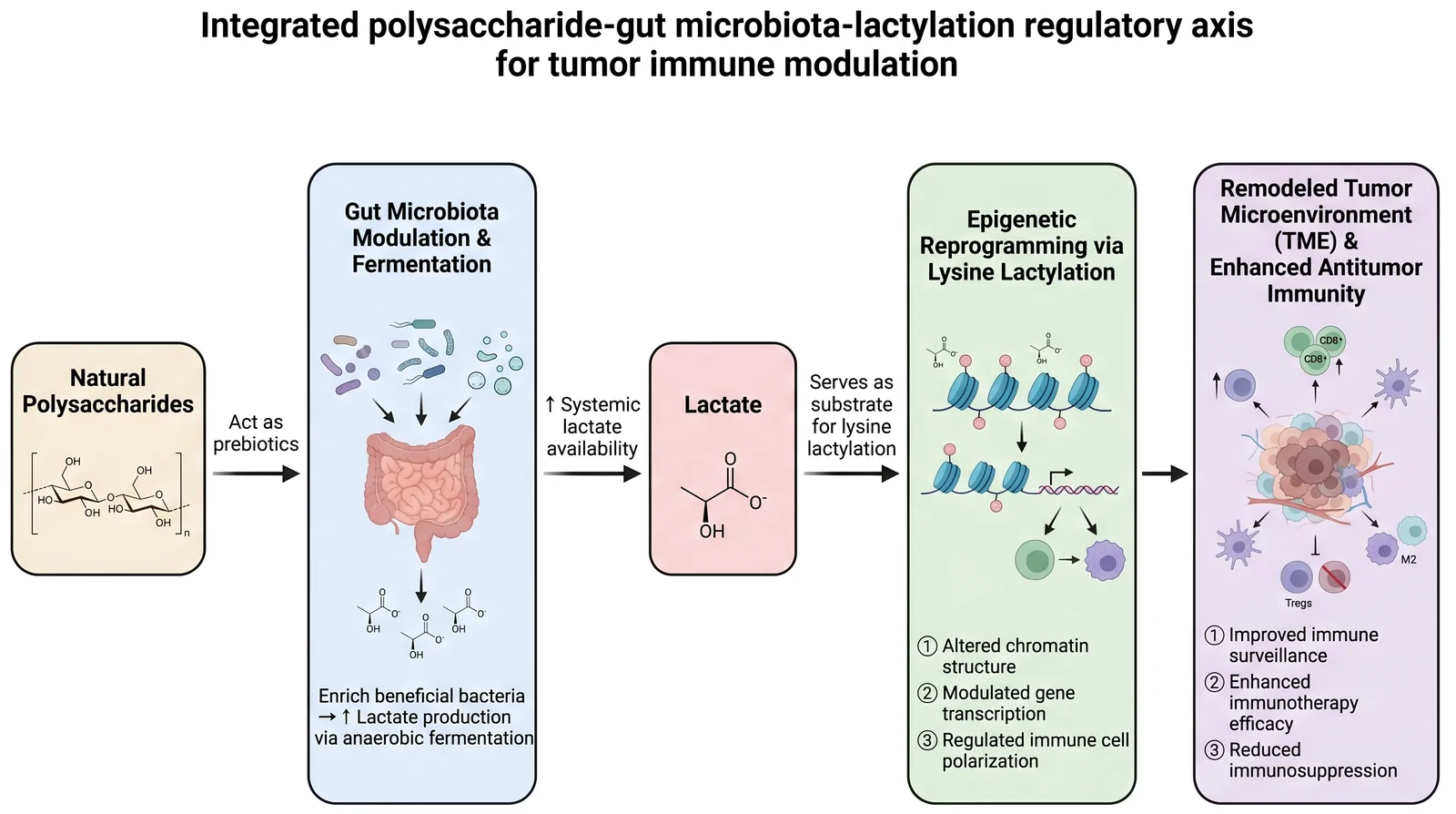
Hypothesized regulatory axis integrating polysaccharide-gut microbiota-lactylation for tumor immune modulation. As prebiotics, certain natural polysaccharides modulate gut microbiota fermentation to increase systemic lactate availability. Lactate drives lysine lactylation and epigenetic reprogramming, which may remodel the tumor microenvironment and enhance anti-tumor immunity in a cell- and tumour-context-dependent manner.

#### 3.3.2 Implications for overcoming immunotherapy resistance

Certain natural polysaccharides have emerged as potent modulators of the tumor immune microenvironment (TME), enhancing immunotherapy efficacy through multifaceted synergistic mechanisms. One key aspect is their ability to activate and enhance immune cell function, thereby boosting antitumor immune responses. Polysaccharides can directly stimulate innate immune cells such as dendritic cells (DCs), macrophages, and natural killer (NK) cells by binding to pattern recognition receptors (PRRs) like Toll-like receptors (*TLRs*) and *Dectin* - 1, triggering downstream signaling pathways including PI3K/Akt, MAPK, and NF-κB. This activation promotes DC maturation, antigen presentation, and subsequent T cell priming, which are critical for effective cytotoxic T lymphocyte (CTL) responses against tumors (49, 57, 239). For instance, polysaccharides derived from *Astragalus membranaceus* (*Astragalus* polysaccharide, APS) have been shown to enhance the activities of macrophages, DCs, NK cells, and T lymphocytes, increasing cytokine secretion and promoting a shift toward a Th1-type immune response, which favors tumor eradication (240). Furthermore, polysaccharides can reprogram tumor-associated macrophages (TAMs) from the pro-tumoral M2 phenotype to the antitumoral M1 phenotype, thus reversing immunosuppression within the TME and facilitating T cell infiltration (28, 241). This immunomodulatory effect is critical for overcoming immune evasion and enhancing the efficacy of immune checkpoint inhibitors (ICIs).

In addition to immune cell activation, polysaccharides serve as effective adjuvants and delivery vehicles in combination immunotherapy. Their physicochemical properties enable the fabrication of nanomedicines and hydrogels that improve the pharmacokinetics and tumor-targeting of immunotherapeutic agents, thereby reducing off-target toxicity and enhancing therapeutic efficacy (57, 65, 177). For example, polysaccharide-based nanoparticles functionalized with tumor antigens and adjuvants can efficiently induce DC maturation and robust antigen-specific humoral and cellular immune responses, resulting in significant tumor growth inhibition in preclinical models (185). Moreover, polysaccharide hydrogels offer biocompatible platforms for sustained release of immunomodulators, further potentiating antitumor immunity (57, 242). The versatility of polysaccharides also allows chemical modifications to enhance stability, bioavailability, and immune recognition, as demonstrated by cationic derivatives that improve macrophage polarization and cytokine secretion (201).

From a translational perspective, combining polysaccharides with immunotherapy holds promising therapeutic potential. Certain natural polysaccharides can sensitize tumors to ICIs by modulating the gut microbiota and metabolic profiles, which are increasingly recognized as critical determinants of immunotherapy response. Studies have shown that polysaccharides from sources such as Chinese yam and sea cucumber can reshape gut microbiota composition, enrich beneficial bacterial taxa, and regulate metabolites that influence immune cell polarization and function, thereby enhancing anti-PD-1/PD-L1 therapy efficacy in colorectal and lung cancer preclinical models (226, 243). This microbiota-mediated mechanism reduces immunosuppressive cell populations like M2 macrophages and regulatory T cells while promoting cytotoxic CD8^+^ T cell infiltration and activation. Additionally, polysaccharides exhibit low toxicity and favorable biocompatibility, making them suitable as adjuncts to conventional immunotherapies to improve safety profiles and reduce adverse effects (244). Beyond well-documented marine and yam-derived polysaccharides, herbal-origin glycyrrhiza polysaccharides also exhibit antitumor immunomodulatory potential. In mouse models, oral glycyrrhiza polysaccharides can reshape gut microbial composition and activate intestinal mucosal TLRs/NF-κB signaling, further promoting the differentiation of intestinal γδT cells toward IFN-γ-producing subsets to restrain tumor progression (245). Nevertheless, such observations remain confined to pre-clinical animal experiments, and further mechanistic and translational investigations are still required.

The clinical translation of polysaccharide-based immunotherapy remains an active area of investigation, with preclinical findings and limited preliminary clinical data suggesting their potential efficacy in combination with immune checkpoint blockade, adoptive cell therapies, and cancer vaccines. For example, APS has been evaluated in clinical trials, with preliminary data suggesting it may enhance immune checkpoint inhibitor responses by modulating immune cell activity and the tumor microenvironment (180). Additionally, polysaccharide-functionalized nanovaccines and delivery systems are being developed to improve antigen presentation and immune stimulation with promising preclinical outcomes (185). The integration of polysaccharides into immunotherapeutic regimens represents a novel strategy to overcome current limitations such as low immunogenicity, immune suppression, and treatment resistance (**Supplementary Figure S10**).

In summary, certain natural polysaccharides enhance immune responses by activating immune cells, remodeling the tumor microenvironment, and modulating gut microbiota, serving as immunomodulators and delivery platforms to improve cancer immunotherapy efficacy and safety. Nevertheless, most mechanistic connections between polysaccharide-reshaped gut microbiota and downstream tumour-intrinsic lactylation remain largely correlative rather than causally proven. Definitive experimental evidence relying on germ-free animal models, antibiotic-mediated microbiota depletion, faecal microbiota transplantation, metabolite-specific inhibition, and stable-isotope tracing remains scarce to fully validate this hypothetical regulatory axis. Future studies combined with nanotechnology will further exploit these beneficial properties and rigorously verify these causal links, laying the groundwork for developing polysaccharide-based combination cancer immunotherapies.

## 4 Current challenges and future perspectives

The crosstalk among polysaccharides, lactylation modifications, and the gut microbiota forms a critical regulatory axis that orchestrates tumor immunity and reshapes the TME. Deepening our mechanistic understanding of this synergistic network will facilitate the development of novel polysaccharide-based strategies to remodel the TME, reverse immunotherapy resistance, and advance cancer immunotherapy.

### 4.1 Current bottlenecks and translational challenges

However, multiple unresolved bottlenecks substantially hinder the clinical translation of polysaccharide-based adjuvant therapy. First, the structural complexity and heterogeneity of natural polysaccharides present a major hurdle, as their immunomodulatory efficacy is highly dependent on structure-activity relationships governed by molecular weight, monosaccharide composition, and chain conformation (201). Elucidating these relationships is essential for rational structural optimization. Meanwhile, non-standardized dosage regimens and poor gastrointestinal bioavailability severely limit the *in vivo* stability and reproducibility of polysaccharide intervention. Second, substantial inter-individual variability in gut microbiota composition leads to heterogeneous therapeutic responsiveness. Importantly, host dietary patterns and clinical antibiotic exposure can profoundly reshape microbial community structure and metabolic output, thereby confounding polysaccharide-mediated TME and immune regulation (238). Third, unified dose-standardization strategies across preclinical models and human studies remain absent, restricting consistent therapeutic validation. Furthermore, high-quality clinical evidence confirming the synergistic efficacy of polysaccharide-ICI combination therapy is still limited. Finally, a comprehensive systems-level interpretation of the polysaccharide–microbiota–lactylation tripartite crosstalk remains insufficient, and integrative multi-omics validation of these complex regulatory networks is still challenging (246). Overcoming these obstacles is critical to promote the clinical translation of polysaccharide-based cancer immunotherapies (**Figure 8**).

**Figure 8:**
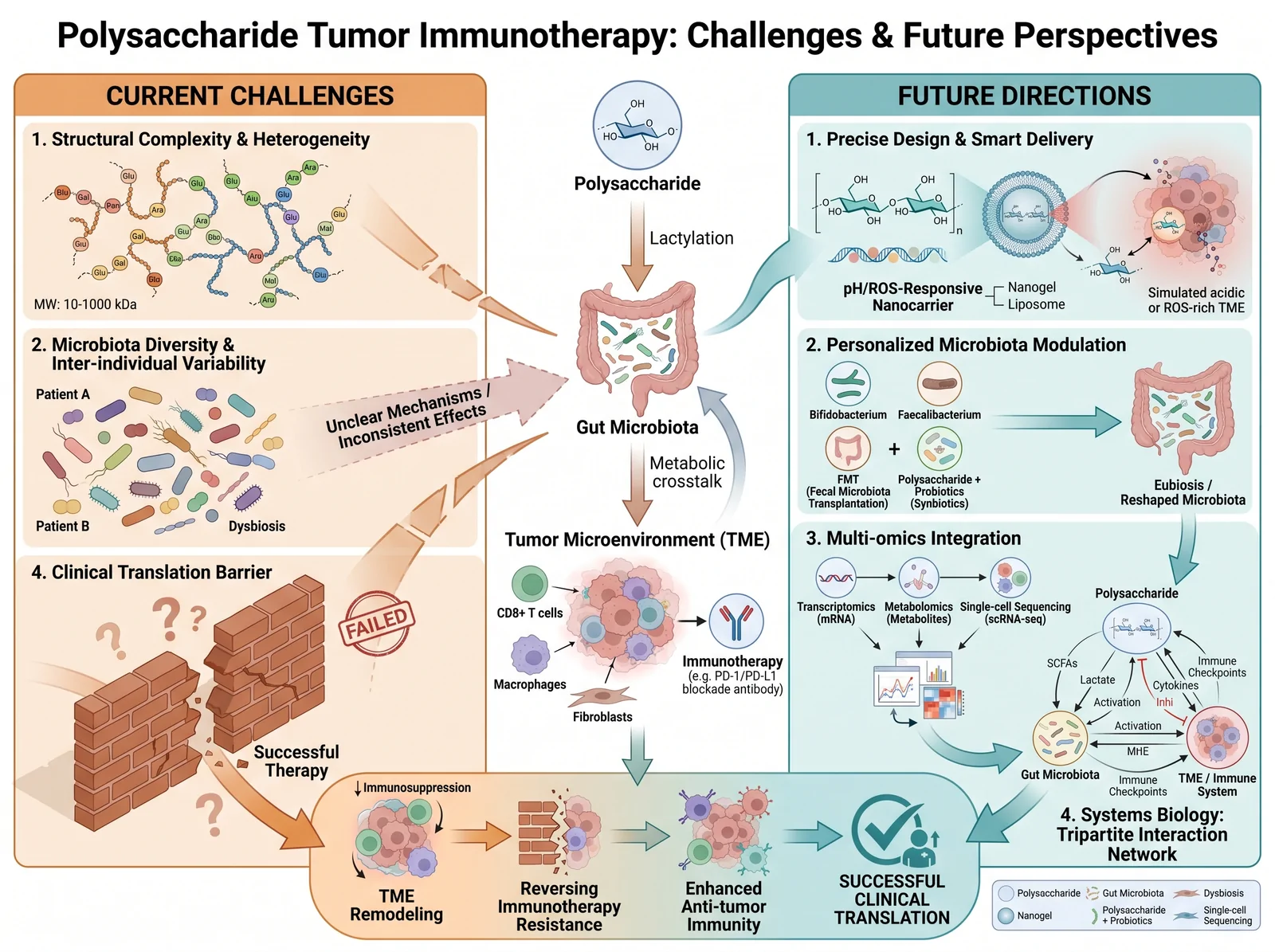
Current challenges and future perspectives of polysaccharide-based cancer immunotherapy. The central axis illustrates how polysaccharides modulate tumor immunity via gut microbiota and lactylation, affecting the TME and immunotherapy response. Key challenges include structural complexity, microbiota variability, unclear crosstalk mechanisms, and clinical translation barriers. Future directions include precise delivery, personalized microbiota modulation, multi-omics integration, and systems biology approaches, aiming to remodel the TME, reverse resistance, and advance clinical translation.

Moreover, inter-individual gut microbiome variability renders therapeutic outcomes highly unpredictable. Host dietary patterns and antibiotic administration can profoundly reshape gut microbial composition and metabolic output, which may confound the immunomodulatory effects of polysaccharide interventions. Another critical barrier lies in the lack of unified dose-standardization schemes across pre-clinical and human studies. To date, high-quality clinical evidence directly validating the anti-tumour adjuvant efficacy of polysaccharides remains scarce, hindering reliable clinical translation.

### 4.2 Proposed experimental strategies to verify the polysaccharide-microbiota-lysine-lactylation hypothesis

As summarized throughout this review, the regulatory axis linking polysaccharides, gut microbiota, and lysine lactylation represents a conceptually integrated but experimentally unvalidated model, which is assembled from discrete observational findings rather than definitive causal evidence. Rigorous multi-layered experimental strategies are therefore required to systematically verify and validate this proposed crosstalk network.

First, germ-free mouse models and antibiotic-induced microbiota-depletion approaches, combined with fecal microbiota transplantation (FMT), constitute the gold-standard toolkit for dissecting microbiota-dependent immunomodulatory mechanisms. Clinical evidence has confirmed that FMT can reshape gut microbial ecology and reverse resistance to immune checkpoint blockade therapy (17). Transferring gut microbiota from polysaccharide-treated donor animals into germ-free recipients enables direct validation of whether microbial remodelling alone is sufficient to recapitulate polysaccharide-triggered alterations in tumour immunity and lactate-dependent epigenetic reprogramming. Selective inhibition of microbial lactate synthesis pathways can further clarify the specific functional contribution of microbial lactate relative to other microbiota-derived metabolites.

Second, *in vivo* stable-isotope metabolic tracing provides a precise technical approach to delineate carbon flux communication between gut microbial fermentation and host epigenetic modification. Isotope-labelled dietary substrates, such as 13C-fibre, have been successfully applied to track microbial carbon incorporation into host histone acetylation in intestinal epithelial cells (247). Adapting this established tracing system will help determine whether gut-microbiota-originated lactate delivers carbon substrates to drive histone and non-histone lysine lactylation within the TME and immune cell compartments. Notably, direct *in vivo* experimental evidence linking microbial lactate production to tumoral and immune lactylation reprogramming remains absent.

Third, comprehensive multi-omics integration, including transcriptomics, proteomics, lactylomics, epigenomics, and metabolomics, is essential to systematically profile dynamic alterations in microbial composition, circulating and tumour-resident metabolite pools, and global lactylation landscapes following polysaccharide intervention (84). Such multi-dimensional profiling enables holistic identification of key regulatory nodes bridging polysaccharide metabolism, microbial remodelling, and epigenetic reprogramming.

Fourth, affinity-enrichment coupled with mass-spectrometry-based lactylome analysis supports site-specific and quantitative detection of dynamic histone and non-histone lactylation modifications in both tumour cells and distinct immune subsets (74). This high-resolution methodology can be applied to precisely characterize polysaccharide-induced lactylation remodelling and identify functionally critical lactylation sites associated with anti-tumour immune regulation.

Finally, prospective clinical cohort studies with multi-omics biomarker profiling are indispensable for translational validation. Longitudinal integration of patient faecal microbiome data, circulating metabolic signatures, and tissue epigenetic profiles can facilitate the identification of microbiota-metabolite-lactylation-based biomarkers capable of predicting individual therapeutic responses to polysaccharide-adjuvant immunotherapy. This clinical-oriented strategy bridges preclinical mechanistic findings and human translational applications.

Collectively, this combination of gnotobiotic animal models, metabolic tracing, multi-omics profiling, site-specific lactylation detection, and clinical biomarker validation enables rigorous discrimination between correlative associations and causal mechanisms, providing systematic verification of the proposed polysaccharide–gut microbiota–lactylation regulatory axis.

### 4.3 Core future research directions

To address the aforementioned translational and mechanistic bottlenecks, future research should prioritize three core directions to accelerate the clinical translation of polysaccharide-based tumour immunotherapy.

#### 4.3.1 Precision polysaccharide structure design and targeted delivery

Rational structural optimization of natural polysaccharides, assisted by high-throughput screening and computational structural modelling, is critical to improve bioactivity, structural stability, and target specificity toward lactate-lactylation immune regulatory pathways. Advanced chemical modification and copolymer conjugation strategies can endow polysaccharides with stimulus-responsive properties, enabling controlled release within the acidic and oxidative TME microenvironment (66, 248, 249). Polysaccharide-based nanogels and pH-cleavable liposomal delivery systems further enhance targeted payload release, thereby optimizing dendritic cell activation and macrophage phenotypic polarization (250). Moreover, integrating polysaccharide scaffolds with lysosomal targeting chimeras represents an innovative strategy to degrade immune checkpoint molecules and alleviate tumour immune evasion (62). Given the emerging role of histone lactylation in driving immunotherapy resistance, developing structure-optimized polysaccharides targeting the lactylation epigenetic network holds great translational potential (251). Scalable standardized synthesis and purification techniques are also urgently required to ensure batch consistency and support future clinical trials.

#### 4.3.2 Personalized gut microbiota modulation

Gut microbiota composition and metabolic function serve as decisive determinants of TME remodelling and ICI therapeutic efficacy (221, 226). Future translational research needs to focus on patient-specific microbial profiling to identify core beneficial microbial taxa and functional metabolites that potentiate anti-tumour immunity and immunotherapy responsiveness (61). Personalized intervention strategies, including targeted probiotic supplementation, dietary polysaccharide prebiotic regulation, and precision FMT, can reshape individual microbial ecosystems to generate synergistic immunotherapeutic effects (226). Mechanistically, gut microbiota-driven macrophage polarization serves as a central link between microbial dysbiosis and TME immunosuppression, while microbial metabolite signalling profoundly remodels tumour metabolic and epigenetic landscapes (252, 253). Multi-omics-based host-microbe interaction analysis will continuously refine personalized polysaccharide adjuvant strategies and facilitate tailored cancer immunotherapy.

#### 4.3.3 Systems-level dissection of the polysaccharide-lactylation-microbiota interaction

Elucidation of the complex tripartite crosstalk between polysaccharides, microbial metabolism, and lactylation modification requires comprehensive systems-level biological strategies. Integrative analysis of transcriptomics, proteomics, epigenomics, and metabolomics can systematically uncover how polysaccharide intervention remodels microbial metabolic profiles, alters lactylation signalling, and reshapes immune cell functional states and tumour progression (221). For instance, transcriptomic profiling has revealed that polysaccharide treatment activates anti-tumour signalling cascades such as p53 and NF-κB, linking metabolic-epigenetic remodelling to TME immunomodulation (251). Furthermore, emerging technologies including single-cell sequencing and spatial transcriptomics enable cell-type-specific mapping of lactylation alterations and immune heterogeneity, precisely distinguishing regulatory effects in tumour cells, macrophages, MDSCs, and T cell subsets. Computational network modelling further helps decode core causal regulatory hubs within this complex axis. This systems-level perspective is essential to resolve current mechanistic ambiguity and guide the rational design of future polysaccharide-based combination immunotherapies.

## 5 Conclusion

Natural polysaccharides represent versatile immunomodulatory agents that remodel the tumour immune microenvironment and improve immunotherapeutic efficacy. Cumulative fragmented evidence demonstrates that polysaccharide intervention, gut-microbiota metabolism and lactate-dependent lysine lactylation each govern antitumour immunity. Synthesizing these scattered observations, this review proposes a conceptual triple regulatory network integrating microbial modulation, metabolic reprogramming and epigenetic modification to synergistically shape antitumour immune responses.

Specifically, bioactive polysaccharides can directly regulate immune-cell function and lactate metabolism while acting as prebiotics to restructure gut microbial communities and reshape metabolite profiles. These combined effects may indirectly alter tumoral lactylation landscapes and immunosuppressive signalling, restoring antitumour immune surveillance and conferring potential tumour-sensitizing effects to immune-checkpoint inhibitors, effects so far largely supported by preclinical findings.

We hypothesize that crosstalk between polysaccharides, gut microbial metabolism and lactylation constitutes an interconnected regulatory axis influencing tumour progression, immune evasion and immunotherapy responses. However, direct experimental proof for the full causal cascade connecting polysaccharide treatment, microbial remodelling and tumour-specific lactylation reprogramming remains absent. Additional bottlenecks include polysaccharide structural heterogeneity, inadequate targeted-delivery approaches and multiple translational obstacles.

In summary, targeting this polysaccharide–gut microbiota–lactylation axis offers an innovative and promising approach to counter immunotherapy resistance. Further mechanistic validation, structural optimization and translational studies are imperative to test this conceptual framework and promote clinical translation of polysaccharide-based cancer immunoadjuvants.

## Supplementary material

Supplementary Figure 1 Natural polysaccharides modulate innate and adaptive immunity via pattern recognition receptors (PRRs). Chitosan binds to the TLR4/MD-2 complex, whereas β-glucans, Ganoderma polysaccharides, and fucoidan serve as ligands for Dectin-1, triggering signaling that activates macrophages, dendritic cells, CD8^+^ T cells, and NK cells, while inhibiting immunosuppressive populations (M2 macrophages, Tregs, MDSCs). This dual regulation enhances anti-tumor immunity and reverses the immunosuppressive tumor microenvironment.

Supplementary Figure 2 Mechanism of Warburg effect-driven lysine lactylation in tumor cells. Enhanced aerobic glycolysis (Warburg effect) in tumor cells promotes lactate production, which serves as a substrate for p300/CBP-mediated lysine lactylation. This modification regulates transcription factors (HIF-1α, c-Myc, p53), driving metabolic reprogramming toward increased glycolysis and suppressed oxidative phosphorylation, thereby forming a positive feedback loop that sustains the Warburg phenotype.

Supplementary Figure 3 Lysine Lactylation-Mediated Regulation of Immune Cell Function in the Tumor Microenvironment. Lactate from tumor cells drives lysine lactylation in the TME, regulating anti-tumor immunity through three pathways: promoting effector function in CD8^+^ T cells, driving macrophage polarization toward an immunosuppressive M2 phenotype, and upregulating immune checkpoints (e.g., PD-L1, B7-H3) via histone lactylation to induce immunotherapy resistance. Targeting the lactate-lactylation axis can restore anti-tumor immunity.

Supplementary Figure 4 Mechanisms of gut microbiota modulation in tumorigenesis and immune evasion. Gut microbiota homeostasis and dysbiosis exert dual effects on tumorigenesis, progression, and immune evasion via microbial metabolites, inflammatory signaling, and immune regulation. Dysbiosis drives immunosuppression and metastasis, while homeostasis enhances anti-tumor immunity and immunotherapy response. Interventions including FMT, probiotics, polysaccharide-based prebiotics, and dietary modification restore balance to reverse immune evasion.

Supplementary Figure 5 Gut microbiota regulation of immunotherapy responses. Gut microbiota composition modulates anti-PD-1/PD-L1 efficacy and immune-related adverse events (irAEs). Responders show high diversity and beneficial taxa promoting anti-tumor immunity, while non-responders display dysbiosis linked to immunosuppression and toxicity. Microbial metabolites, immune signaling, and interventions (FMT, probiotics, prebiotics) regulate these effects, with restoration of homeostasis enhancing therapy outcomes.

Supplementary Figure 6 Some natural-polysaccharide-mediated regulation of lactate metabolism and lysine lactylation. Certain natural polysaccharides regulate lactate metabolism and lysine lactylation via three upstream pathways: modulating glycolysis in tumor/immune cells, targeting transporters/enzymes (MCT4/LDHB), and enriching lactate-producing gut microbiota. These actions increase systemic lactate pools, which are converted to lactyl-CoA for histone/non-histone lactylation, ultimately driving epigenetic reprogramming and TME remodeling.

Supplementary Figure 7 Some natural-polysaccharide-mediated regulation of lactylation modulates tumor-associated immune cell function. Certain natural polysaccharides reduce lactate accumulation and excessive protein lactylation in the TME, reshaping key immune cell functions: enhancing cytotoxic activity of CD8^+^ T and NK cells, repolarizing M2 macrophages to the anti-tumor M1 phenotype, and attenuating immunosuppression in MDSCs and Tregs via reduced lactylation of Arg1 and FOXP3.

Supplementary Figure 8 Regulation of gut-microbiota-derived metabolites and downstream effects mediated by certain natural polysaccharides. Certain natural plant and fungal polysaccharides modulate beneficial gut microbiota to promote production of SCFAs and lactate via fermentation. SCFAs regulate immunity and intestinal barrier function, while lactate maintains gut homeostasis and serves as a key substrate for protein lactylation and epigenetic reprogramming in tumor cells.

Supplementary Figure 9 Some natural-polysaccharide-mediated modulation of gut microbiota for antitumor immunity. Certain natural polysaccharides act as prebiotics to enrich beneficial gut bacteria and regulate microbial metabolites, leading to TME remodeling and enhanced antitumor immunity, including synergism with anti-PD-1 immunotherapy. The schematic also depicts verification approaches (microbiota depletion, FMT) and advanced strategies (polysaccharide-decorated extracellular vesicles).

Supplementary Figure 10 Synergistic mechanisms by which certain natural polysaccharides enhance cancer immunotherapy efficacy. Certain natural polysaccharides exert three main effects: direct immune activation via pattern recognition receptors (e.g., TLRs, *Dectin* - 1), serving as delivery vehicles/adjuvants, and sensitizing tumors to immunotherapy through gut microbiota modulation. These combined actions reverse immunosuppression, improve tumor targeting, and enhance the response to immune checkpoint inhibitors.

Supplementary Table 1 Summary of key evidence for polysaccharides regulating lactylation and gut microbiota to enhance cancer immunotherapy.
