# Supplementary material for: Polysaccharide-mediated modulation of gut microbiota and lysine lactylation: implications for tumor microenvironment remodeling and cancer immunotherapy
Source: Front Immunol. 2026 Oct 1;17:1924150. doi: 10.3389/fimmu.2026.1924150 (PMC13670173; doi:10.3389/fimmu.2026.1924150)
Supplement: Supplementary file 1 [file Table1.docx]

| Table S1. Summary of Key Evidence for Polysaccharides Regulating Lactylation and Gut Microbiota to Enhance Cancer Immunotherapy | | | | | | |
| --- | --- | --- | --- | --- | --- | --- |
| **NO** | **Polysaccharide Type (Source/Structure)** | **Research Model (Cell/Animal/Clinical)** | **Gut Microbiota Modulatory Effects (Specific Taxa/Diversity)** | **Lactylation Regulation Evidence (Molecular/Protein/Level Changes)** | **Immunotherapy Association (Synergy with ICIs/Efficacy Indicators)** | **References** |
| 1 | Representative Plant Polysaccharides: Astragalus polysaccharides (APS/PG2: Glc/Gal/Rha/Ara; 12–135 kDa); Belapectin (Galectin-3 inhibitor); Apple polysaccharides (AP: GalA/Gal; 5–10 kDa) | Cell (RAW264.7, BMDM); Animal (C57BL/6, 4T1/LLC tumor models); Clinical (NSCLC, MM, HNSCC) | Indirect relevance (APS/ *Dendrobium officinale* polysaccharides modulate gut microbiota; no specific taxa reported) | No direct lactylation data; indirectly linked to TME metabolic balance via cytokine (TNF-α, IL-6, IL-12) and NO/ROS regulation | Repolarizes M2→M1 macrophages (TLR/NF-κB/MAPK pathways); synergizes with ICIs (PD-1 inhibitors) and chemotherapy; inhibits tumor growth; enhances CD8^+^ T cell activity; clinical efficacy in NSCLC/MM/HNSCC | 28. Wei et al. 2024 |
| 2 | Lentinan (LNT): β-(1→3)-D-glucan with β-(1→6) branches from *Lentinus edodes*; clinical biological response modifier (BRM) | Cell (RAW264.7, CTL, NK assays); Animal (syngeneic tumor models: Meth‑A, Colon‑26, YAC‑1, B16); Clinical (NSCLC patients, gastric cancer) | No direct gut microbiota modulation data reported in this review | No direct lactylation data; indirectly linked to TME metabolic balance via cytokine regulation | Enhances CTL/NK cell cytotoxicity; restores suppressed killer cell activity; reduces Treg proportion; shifts Th2 to Th1 (↑IFN-γ, TNF-α, IL-12; ↓IL-10, TGF-β1); synergizes with trastuzumab/gemcitabine; improves patient survival/QoL | 47. Li et al. 2021 |
| 3 | Astragalus Polysaccharides -based Nanovaccines (APS-NVs): Low molecular weight (12.19 kDa, optimal); self-adjuvant + carrier; main monosaccharide (Glc >90%); CaP-coated for stability | Cell (BMDCs, B16-OVA, LLC cells); Animal (C57BL/6 mice bearing B16-OVA melanoma/LLC; nude mice); Preclinical (therapeutic tumor models) | Indirect relevance (APS modulates gut microbiota in prior studies; no specific taxa reported) | No direct lactylation data; indirectly linked to TME metabolic balance via cytokine (IL-12p70, TNF-α, IFN-γ) regulation | Enhances DC maturation (TLR4/TLR2/dectin-1-mediated); targets lymph nodes; promotes CD8^+^ T cell responses and immune memory; inhibits tumor growth (B16-OVA/LLC); superior to OVA+Alum; no systemic toxicity | 55. Li N, et al.  2024 |
| 4 | Diverse natural plant & fungal polysaccharides (*Astragalus, Lycium, Ganoderma, Portulaca, Angelica, Antrodia* etc., variable MW & glycosidic linkages, multi-type heteropolysaccharides) | Cell (BMDCs, moDCs, tumor cell lines including CT26, 4T1, B16); Animal (BALB/c, C57BL/6 mice with melanoma, colon, breast cancer models); Clinical (NSCLC, multiple myeloma, prostate, liver cancer cohorts) | Indirect relevance; individual polysaccharides reported to alter intestinal flora in separate studies; no specific bacterial taxa characterized within this review | No direct lactylation-related experimental data; regulates TME metabolism indirectly through DC-derived pro-inflammatory cytokines (IL-12p70, TNF-α, IFN-γ) and immune cell metabolic reprogramming | Activates DC maturation via TLR2/TLR4/Dectin-1/NF-κB/MAPK/Notch pathways; elevates MHC-I/II & co-stimulatory molecules (CD80/CD86); boosts CD4+/CD8+ T cell proliferation & Th1 polarization; enhances DC vaccine, DC-CIK, adoptive cell and anti-PD-1 immunotherapy efficacy; restrains primary/metastatic tumor progression | 56. Xiong H, et al. 2023 |
| 5 | Representative Polysaccharide Hydrogels: Hyaluronic acid (HA); Alginate; Chitosan; Dextran (natural polysaccharides, crosslinked into hydrogels) | Cell (DCs, CAR-T cells, tumor cells); Animal (B16F10, CT26, hepatocellular carcinoma models); Clinical (breast cancer, wound healing) | Indirect relevance (chitosan/alginate/HA modulate gut microbiota; no specific taxa reported) | No direct lactylation data; indirectly linked to TME metabolic balance via T cell activation and cytokine (IL-12, TNF-α) regulation | Delivers CAR-T cells/ICIs/vaccines; enhances T cell infiltration and DC maturation; synergizes with PD-1/CTLA-4 inhibitors; inhibits tumor growth; reduces systemic toxicity | 57. Zhou Y, et al. 2025 |
| 6 | *Coriolus versicolor* Polysaccharide (CVPW-1): 1.03×10⁶ Da; mannose-glucose-galactose heteropolysaccharide with (1→3/6)-linked glucan backbone | Cell (RAW264.7 macrophages, 4T1 tumor cells); Animal (BALB/c mice bearing 4T1 breast cancer) | No direct gut microbiota regulation data | No direct lactylation evidence; indirectly linked to TME metabolic-immune crosstalk via macrophage polarization and inflammatory signaling | Reverses M2→M1 tumor-associated macrophage (TAM) polarization; suppresses TME immunosuppression; enhances anti-tumor immune response; no ICI synergy data reported in this study | 58. Bi S, et al. 2024 |
| 7 | *Pholiota adiposa* Polysaccharide (PAP-1a): 16.453 kDa; monosaccharide composition (mannose/arabinose/galactose/xylose, etc.); coated on selenium nanoparticles (PAP-SeNPs) | Cell (RAW264.7, Hepa 1–6, H22 cells); Animal (H22 hepatoma-bearing C57BL mice); Preclinical (anti-hepatoma model) | Indirect relevance (Pholiota adiposa polysaccharides modulate gut microbiota; no specific taxa reported) | No direct lactylation data; indirectly linked to TME metabolic balance via M1 macrophage polarization and cytokine (TNF-α, IL-12p40) regulation | Reverses M2→M1 TAM polarization (Tlr4/Myd88/NF-κB pathway); enhances CD3+CD4+/CD8+ T cells; inhibits hepatoma growth; no systemic toxicity | 59. Xu et al. 2024 |
| 8 | Sea Cucumber Polysaccharides (SCP): Main components = fucosylated chondroitin sulfate + fucoidan sulfate; sulfated polysaccharides | Cell (CT26 CRC cells); Animal (CT26 tumor-bearing Balb/C mice); Preclinical (anti-PD1 resistance model) | Direct modulation: ↑ *Bifidobacterium pseudolongum* and *Faecalibaculum rodentium*; ↑ microbial diversity (ACE index) | No direct lactylation data; indirectly linked to TME metabolic balance via microbiota-mediated T cell activation | Synergizes with anti-PD1 to overcome immunotherapy resistance; reduces tumor volume by 42.78%; ↑ IFN-γ+ CD8+ T cells; ↓ regulatory CD4+ T cells; effect mediated by gut microbiota/FMT | 61. Li J, et al. 2025 |
| 9 | Polyporus Polysaccharide (PPS): Derived from *Polyporus umbellatus*; immunomodulatory; used as microneedle (MN) matrix | Cell (B16F10, EMT6, MCF7-TR cells; BMDMs, THP-1 macrophages); Animal (B16F10/EMT6 tumor-bearing mice); Preclinical (synergistic immunotherapy model) | Indirect relevance (Polyporus polysaccharides modulate gut microbiota; no specific taxa reported) | No direct lactylation data; indirectly linked to TME metabolic balance via M1 macrophage polarization and CD47 degradation | Synergizes with CD47-LYTAC (RS17- M6P_3_); degrades CD47; reprograms M2→M1 TAMs (NF-κB/NLRP3 pathway); enhances CD4^+^/CD8^+^ T cell infiltration; synergizes with anti-PD-L1; no systemic toxicity | 62. Lin J, et al. 2025 |
| 10 | *Menispermum dauricum* DC. Polysaccharide (MDP): 6.16 kDa; (1→6)-α-D-glucan main chain | Cell (RAW264.7, Lewis-LUC lung cancer cells); Animal (C57BL/6J Lewis tumor-bearing mice) | No direct gut microbiota modulation data | No direct lactylation data; indirectly linked to TME balance via M1 polarization | Polarizes M2→M1 TAMs; increases CD3+/CD8+ T cells; promotes Th1 polarization; alleviates tumor immunosuppression; ameliorates CTX-induced organ injury; synergizes with chemotherapy | 63. Yang P, et al. 2024b |
| 11 | *Crocus sativus petal* polysaccharide (PCSPB)*;* reconstructed tumor microenvironment | Cell (S180, HCT116, A549, UM-UC3); Animal (C57BL/6 mice bearing S180 sarcoma) | No direct gut microbiota modulation data | No direct lactylation data; indirectly linked to TME remodeling via immune cell regulation | Reprograms M2→M1 TAMs; increases CD8^+^ T/NK cells; reduces Tregs/MDSCs/ TAMs; inhibits tumor growth; low toxicity | 64. Tu J, et al. 2023 |
| 12 | Representative Stimulus- Responsive Polysaccharides: Hyaluronic acid (HA); Chitosan; Heparin; Dextran; Cyclodextrin; Alginate; Fucoidan (stimulus- responsive, modifiable, biocompatible) | Cell (4T1, B16F10, CT26 cells; DCs, TAMs); Animal (4T1/B16F10/CT26 tumor-bearing mice); Preclinical (combination immunotherapy models) | Indirect relevance (chitosan/fucoidan/ginseng polysaccharides modulate gut microbiota; no specific taxa reported) | No direct lactylation data; indirectly linked to TME metabolic balance via T cell activation, TAM polarization, and cytokine (IL-12, TNF-α, IFN-γ) regulation | Synergizes with chemotherapy/PDT/PTT/ICIs; induces ICD; repolarizes M2→M1 TAMs; enhances CD4+/CD8+ T cell infiltration; inhibits tumor growth/metastasis; reduces systemic toxicity | 65. Li Q, et al. 2023b |
| 13 | Representative Tumor Microenvironment-Responsive Polysaccharides: Hyaluronic acid (HA); Chitosan; Dextran; Alginate; Heparin; Cyclodextrin; Starch; Cellulose (pH/GSH/ROS /enzyme/ hypoxia-responsive, biodegradable, modifiable) | (Cell/Animal/Clinical): Cell (4T1, B16F10, CT26, MCF-7, RAW264.7); Animal (murine tumor models); Preclinical (TME-responsive delivery systems) | Indirect relevance (chitosan, alginate, starch modulate gut microbiota; no specific taxa reported) | No direct lactylation data; indirectly linked to TME metabolic reprogramming via immune cell activation and redox balance | Delivers chemotherapeutics/ PDT/PTT/ICDs/ICIs; remodels TME; repolarizes M2→M1 TAMs; enhances CD8+ T infiltration; inhibits tumor growth/metastasis; reduces systemic toxicity | 66. Wu Q, et al. 2023 |
| 14 | Chitosan/Fucoidan Polysaccharide Nanoparticles (CS-Arg/Fuc-Bio): Biotinylated marine polysaccharide NPs; dual-targeting (P-selectin/biotin receptor); pH-responsive | Cell (HCT116, HT29, HUVEC, RAW264.7); Animal (BALB/c nude mice bearing HT29 colorectal cancer) | No direct gut microbiota data | No direct lactylation data; indirectly linked to TME reprogramming via ICD and M1 macrophage polarization | Induces immunogenic cell death (ICD); repolarizes M2→M1 TAMs; enhances CD8^+^ T cells; inhibits angiogenesis/ glycolysis; synergizes with chemotherapy; good biosafety | 67. Wang H, et al. 2026 |
| 15 | Representative Stimulus-Responsive Polysaccharides: Hyaluronic acid (HA); Chitosan; Heparin; Dextran; Cyclodextrin; Alginate; Fucoidan (biocompatible, modifiable, stimulus-responsive) | Cell (4T1, B16F10, CT26, MCF-7 cells; DCs, TAMs, macrophages); Animal (4T1/B16F10/CT26 tumor-bearing mice); Preclinical (combination immunotherapy models) | Indirect relevance (chitosan/fucoidan/ginseng polysaccharides modulate gut microbiota; no specific taxa reported) | No direct lactylation data; indirectly linked to TME metabolic balance via T cell activation, TAM polarization, and cytokine (IL-12, TNF-α, IFN-γ) regulation | Achieves synergistic chemo-/photo-/immunotherapy via stimulus-triggered drug release; induces ICD; repolarizes M2→M1 TAMs; enhances CD4+/CD8+ T cell infiltration; inhibits tumor growth/metastasis; reduces systemic toxicity. | 68. Liu W, et al. 2024 |
| 16 | Low-Methoxyl Pectin (LMP)-armored nanoemulsion (LMP@EL-CNE): Polysaccharide-coated colon-targeted nanoemulsion; pH/microflora-responsive | Cell: CT26.WT (ferroptosis/ uptake)  Animal: BALB/c mice (orthotopic CT26 CRC model)  Preclinical: oral colon-targeted chemo-immunotherapy | Direct modulation: restores α-diversity; ↓ *Escherichia*; normalizes *Akkermansia*; ↑ *Ligilactobacillus*; repairs barrier (ZO-1/Occludin) | No direct lactylation data; triggers ferroptosis (↓GPX4/SLC7A11/GSH, ↑MDA/ROS/lipid ROS) via mitochondrial dysfunction | Creates "hot" TME: ↑ mature DCs & CD8+ T cells; ↑ IFN-γ/TNF-α; ↓ PD-1/Tim-3 exhaustion; inhibits tumor growth; prolongs survival; low toxicity | 69. Zhu Q, et al. 2026 |
| 17 | FAY combination:  *Laminaria polysaccharide* (FU): Mw= 27,713 Da; Aloe polysaccharide (AL): Mw= 44,425 Da; Yeast β-glucan (YG): Mw=29,296 Da;  FAY combination: FU:AL:YG= 0.8:0.2:1 | In vitro (lactic acid bacterial fermentation; feline/canine in vitro digestion; feline/canine fecal fermentation); 16S rRNA sequencing + metabolomics | Direct evidence: Enriches beneficial taxa (*Bifidobacterium, Lactobacillus, Megamonas*, Prevotella_9); suppresses harmful bacteria (*Fusobacterium*, *Desulfovibrio*); restores microbiota diversity; balances *Firmicutes/Bacteroidota* ratio; FAY combination shows strongest modulatory effects | Indirect evidence: No direct lactylation data; promotes SCFAs (butyric/propionic acid) and lactic acid; reduces odor metabolites; regulates bile secretion/arachidonic acid metabolism | Indirect evidence: SCFA production and gut microbiota balance correlate with improved gut immune homeostasis; beneficial taxa (Bifidobacterium, Lactobacillus) support antitumor immunity framework | 166. Bai H, et al. 2025 |
| 18 | Tucum-do-cerrado polysaccharides (TUC): soluble dietary fiber mixture (glucuronoarabinoxylan + glucomannan), Mw=70 kDa; mannose/xylose/glucose/uronic acids (26.5:12.0:11.3:27.5) | In vivo (Ehrlich carcinoma-bearing Swiss mice); ex vivo (mouse liver perfusion); histology + biochemical assays | Indirect evidence: No direct microbiota data; prior reports of arabinoxylan modulating gut microbiota and SCFA production | Indirect evidence: No direct lactylation data; modulates oxidative stress (increases LPO, decreases GSH) and inflammation (TNF-α/IL-1β/NO) in tumor microenvironment | Direct evidence: Exerts antineoplastic effects (reduces tumor weight/volume, induces necrosis) in mammary cancer model; synergizes with anti-tumor inflammation; no hepatotoxicity, supporting safety for combined therapy | 169. de Oliveira KM, et al. 2024 |
| 19 | Francisella tularensis O-antigen capsule (FTT capsule); bacterial polysaccharide, repeating tetra-saccharide structure with amine-capped core | In vitro (murine bone marrow-derived macrophages, BMdMs); FTT infection model | Indirect evidence: Conserved short-chain carbon (lactate) exploitation mechanism parallel to gut microbiota-SCFAs crosstalk | Indirect evidence: Upregulates MCT4 and LDHB to enhance lactate oxidation into the TCA cycle in infected macrophages; **lysine lactylation was not measured in the study | Indirect evidence: Inhibits pro-inflammatory cytokines in macrophages; this is a bacterial virulence factor study, not an antitumour polysaccharide | 170.Jessop F, et al. 2025 |
| 20 | Chitosan (cationic, pH- responsive), hyaluronic acid (CD44-targeting), dextran (biocompatible), alginate (mucoadhesive), *Ganoderma lucidum* polysaccharides | In vitro (DCs, T cells, macrophages); in vivo (tumor-bearing mice); preclinical | Indirect evidence: Polysaccharides (dextran/alginate) act as prebiotics to modulate gut microbiota diversity; may enhance SCFA production (established in polysaccharide research) | Indirect evidence: Modulates metabolic reprogramming (e.g., ROS, glycolysis) and immune cell activation, potentially influencing lactate metabolism and lactylation (no direct modification data) | Review-summarized evidence: Summarizes DC maturation, CD8^+^ T cell proliferation, M1 macrophage polarization, and potential synergy with anti-PD-1 reported in original studies. | 177. Zeng Y, et al. 2021 |
| 21 | Multiple TCM polysaccharides: APS (*Astragalus membranaceus*, glucose/galactose/arabinose), GLP (*Ganoderma lucidum*, β-glucan backbone), LNT (*Lentinus edodes*, β-(1→3)-glucan), BSP (Bletilla striata, α/β-mannose/glucose) | In vitro (DCs, macrophages, T cells); in vivo (tumor-bearing mice: CRC, NSCLC, breast cancer); clinical (NSCLC, gastric cancer, esophageal cancer) | Indirect evidence: GLP/APS reshapes gut microbiota diversity, increases beneficial taxa (e.g., Parabacteroides distasonis), and modulates SCFA production | Indirect evidence: Regulates glycolysis and lactate metabolism, inhibits lactate-induced immunosuppression, potentially influencing lactylation (no direct modification data) | Direct evidence: Enhances ICI efficacy (APS/GLP + anti-PD-1); activates DC/T cell/macrophage- mediated immunity; nano-formulated polysaccharides (GLP- APBA-MTX NPs) improve chemo-immunotherapy synergy; clinical data shows reduced chemotherapy toxicity and improved quality of life | 184. Sun L, et al. 2025 |
| 22 | Benzoylated inulin (InBz): Modified polysaccharide (from *dahlia* inulin), β-(2→1)-linked polyfructosyl units, benzoyl substitution (degree of substitution: 3.89), TLR4 agonist | In vitro (DC2.4, RAW264.7, THP-1 cells); in vivo (E.G7-OVA tumor-bearing mice) | Indirect evidence: Parent inulin modulates gut microbiota diversity and SCFA production; enhances beneficial taxa (established prebiotic effect) | Indirect evidence: Regulates TLR4-mediated immune cell activation and glycolysis, potentially influencing lactylation (no direct modification data) | Direct evidence: Functions as cancer vaccine adjuvant; induces potent CTL responses (specific killing >93%) and humoral immunity; InBz-OVA NPs inhibit tumor growth (81% volume reduction) and enhance CD4^+^/CD8^+^ T/NK cell infiltration | 185. Cui Z, et al. 2025 |
| 23 | *Polygonatum kingianum* polysaccharides (PKP60/PKP70/PKP80; neutral polysaccharides with triple-helix structure, Mw: 3.19–19.58 kDa) | In vitro (human fecal microbiota fermentation) | Direct evidence: PKP70 enriches *Bifidobacterium*; PKP60 promotes *Anaerostipes/Holdemanella*; all fractions increase SCFA-producing taxa | Indirect evidence: No direct lactylation data; SCFAs (butyrate) act via HDAC inhibition (known to enhance T cell function) | Indirect evidence: SCFA production (butyrate/propionate) correlates with improved ICI efficacy; *Bifidobacterium* enrichment supports antitumor immunity | 198. Zhang N, et al. 2024 |
| 24 | *Oudemansiella raphanipes* polysaccharides (OrPs); fungal polysaccharides, total sugar content 97.26%, monosaccharide composition: mannose/rhamnose/glucose/xylose | In vivo (Kunming mice) | Direct evidence: OrPs enriches *Lachnospiraceae/Faecalibaculum/Bifidobacterium*; reduces potentially pathogenic bacteria; alters gut microbiota structure | Indirect evidence: No direct lactylation data; SCFAs (butyric/acetic acid) enhance intestinal barrier and T cell function | Indirect evidence: SCFA production correlates with improved ICI efficacy; probiotic enrichment (*Bifidobacterium*) supports antitumor immunity | 199. Fang Q, et al. 2023 |
| 25 | *Cyclocarya paliurus* polysaccharide (CPP): leaf-derived heteropolysaccharide (total sugar 64.8%, uronic acid 23.5%, MW 900 kDa; composed of arabinose, rhamnose, galactose) | In vivo (healthy Kunming mice; 50/100/200 mg/kg, 20 days) | Direct evidence: ↑ α-diversity (Chao1, Shannon); ↑ *Lachnospiraceae, Clostridiales, Clostridia, Alistipes*; ↓ *Lactobacillus, Bacteroides, Proteobacteria;* ↑ SCFAs (acetate/propionate/butyrate/valerate) | Indirect evidence: SCFAs modulate immune metabolism and may affect lactylation via HDAC inhibition and energy metabolism reprogramming; no direct lactylation data | Direct evidence: Improves gut microbiota metabolic function (starch/sucrose, amino acid, glycerolipid metabolism); enhances intestinal homeostasis and immune microenvironment | 200.Wu T, et al. 2021b |
| 26 | *Lepidium meyenii* Polysaccharide (MPW): Mw = 8684.5 Da; main chain →4)-α-D-Glcp-(1→, branched via O-6 bond; cationic derivative (C-MPW, DS = 53.37%) | Cell (RAW264.7, 4T1 cells); Animal (4T1 tumor-bearing BALB/c mice); Preclinical (synergistic chemo- immunotherapy model) | Indirect relevance (Lepidium meyenii polysaccharides modulate gut microbiota; no specific taxa reported) | No direct lactylation data; indirectly linked to TME metabolic balance via M1 macrophage polarization and cytokine (IL-12, TNF-α, IFN-γ) regulation | Reverses M2→M1 TAM polarization (NF-κB/STAT1/STAT3 pathways); synergizes with Dox; inhibits tumor growth/metastasis; reduces systemic toxicity; C-MPW shows stronger efficacy than MPW | 201. Guo T et al. 2020 |
| 27 | *Fritillaria* polysaccharides (FPS): Polysaccharides from 10 *Fritillaria* species (mainly glucan; MW 2.89×10⁶–6.75×10⁶ kDa; monosaccharides: Glc, Gal, Ara, Xyl, Rha, GalA) | In vivo (C57BL/6J mice; 200 mg/kg, 14 days) | Direct evidence: ↑ α-diversity; ↑ *Akkermansia, Bifidobacterium, Lachnospiraceae*; ↓ *Faecalibaculum, Parabacteroides*; modulates *Firmicutes/Bacteroidota* ratio | Indirect evidence: Modulates SCFA-producing taxa and immune-related microbiota, potentially affecting lactylation and tumor immune microenvironment | Direct evidence: Improves intestinal homeostasis; exerts potential effects against gastrointestinal and inflammatory diseases; supports immune microenvironment regulation | 202.Wei W, et al. 2023 |
| 28 | Acacia gum polysaccharide (AG): branched arabinogalactan from *Acacia senegal* & *Acacia seyal*; Ara/Gal/GlcA; Rha terminal | In vitro (pH-controlled human colon model; fecal microbiota fermentation) | Direct evidence: selectively ↑ *Bifidobacterium, Lactobacillus, Atopobium*; inhibits *Clostridium histolyticum* group; ↑ SCFAs (acetate/propionate/butyrate) | Indirect evidence: SCFAs modulate immune metabolism and may affect lactylation via HDAC inhibition and energy metabolism reprogramming | Direct evidence: Exerts prebiotic effects; improves gut homeostasis; suppresses pathogenic microbiota and gut dysbiosis | 203.Rawi MH, et al. 2021 |
| 29 | *Ophiopogon japonicus* polysaccharide (OJTP): Ophiopogon japonicus-derived bioactive polysaccharide (total polysaccharide content 52.79%) | In vivo (bleomycin-induced pulmonary fibrosis mice; 50/120/300 mg/kg, 21 days) | Direct evidence: Remodels gut microbiota; enriches *Muribaculaceae bacterium, Duncaniella muricolitica, Prevotella* sp. MGM2; upregulates polysaccharide-degrading CAZymes (GH13, GH28, CBM13) | Indirect evidence: Modulates urobilinogen, 5-AVAB and amino acid/porphyrin metabolism; indirectly affects immune-metabolic reprogramming and potential lactylation | Direct evidence: Ameliorates pulmonary fibrosis; reduces collagen deposition and α-SMA expression; restores gut-lung axis homeostasis | 204. Zhang PP, et al 2026 |
| 30 | Fermented Astragalus polysaccharides (FAPS); fermented by *Lactobacillus rhamnosu*s, optimized fermentation parameters | In vitro (human fecal microbiota fermentation) | Direct evidence: FAPS enriches *Lactobacillus/Faecalibacterium/Blautia*; inhibits *Shigella/Clostridium_sensu_stricto_*1; increases microbial diversity | Indirect evidence: No direct lactylation data; SCFAs (acetic/propionic acid) enhance intestinal barrier and T cell function | Indirect evidence: SCFA production correlates with improved ICI efficacy; probiotic enrichment (Lactobacillus) supports antitumor immunity | 208.Yang P, et al. 2024 |
| 31 | Degraded *Acanthopanax senticosus* polysaccharides (ASPS-1); degraded by H_2_O_2_-Vc, Mw: 4288 Da, anti-digestive | In vitro (broiler fecal microbiota fermentation) | Direct evidence: ASPS-1 enriches *Faecalibacterium/Paraprevotella*; inhibits *Fusobacteria*; increases microbial diversity | Indirect evidence: No direct lactylation data; SCFAs (butyric/acetic acid) enhance intestinal barrier and T cell function | Indirect evidence: SCFA production correlates with improved ICI efficacy; probiotic enrichment (Faecalibacterium) supports antitumor immunity | 209.Wang X, et al. 2024 |
| 32 | Sulfated modified *Poria cocos* polysaccharide (PCY-S2); sulfated derivative of alkali-soluble polysaccharide, degree of substitution: 0.59 | In vitro (human fecal fermentation); In vivo (KM mice) | Direct evidence: In vitro—↑ *Streptococcus/Bifidobacterium/Lactococcus*; In vivo—↓ F/B ratio, ↑ *unclassified_f_Muribaculaceae/Prevotellaceae_UCG-001/Alloprevotella* | Indirect evidence: No direct lactylation data; SCFAs (butyric/propionic acid) and reduced IL-6/TNF-α enhance intestinal barrier and T cell function | Indirect evidence: SCFA production and anti-inflammatory effects correlate with improved ICI efficacy; probiotic enrichment (Bifidobacterium) supports antitumor immunity | 210. Ma L, et al. 2025 |
| 33 | *Auricularia auricula* polysaccharides (AAP); fungal polysaccharides, total carbohydrate content 85.2%, GlcA/Man/Xyl/Glc | In vitro (human fecal fermentation) | Direct evidence: AAP enriches *Bacteroides/Parabacteroides/Lachnoclostridium*; inhibits *Bilophila/ Morganella/Escherichia-Shigella*; ↑ *Bacteroidota/Firmicutes* ratio | Indirect: No direct lactylation; SCFAs (butyrate/acetate) & regulated Trp/bile acid pathways enhance barrier & T cell function | Indirect evidence: SCFA production and metabolic regulation correlate with improved ICI efficacy; probiotic enrichment (Parabacteroides) supports antitumor immunity | 211. Liu N, et al. 2025a |
| 34 | Crude Stellariae Radix polysaccharides (CSRPs); plant polysaccharides, molecular weight: 31,309 Da, monosaccharide composition: Gal/Glc/ Xyl/ Fru/ Man/ Rha | In vitro (probiotic culture); In vivo (ICR mice) | Direct evidence: In vitro—promotes Lactobacillus acidophilus / Bifidobacterium longum; In vivo- enriches Dubosiella/ Faecalibaculum/ Bifidobacterium; inhibits *Bacteroides /Enterorhabdus*; reduces Firmicutes/ Bacteroidota ratio | Indirect evidence: SCFA-related microbial regulation correlates with improved ICI efficacy; beneficial taxa (Bifidobacterium) support antitumor immunity | Indirect evidence: SCFA-related microbial regulation correlates with improved ICI efficacy; beneficial taxa (Bifidobacterium) support antitumor immunity | 212. Wang H, et al. 2023 |
| 35 | Longan pulp polysaccharide (LPIIa); fruit-derived polysaccharide, molecular weight: 159.3 kDa, arabinogalactan structure | In vitro (human fecal fermentation, Caco-2/ RAW264.7 co-culture); in vivo (BALB/c mice, cyclophosphamide-induced intestinal injury model) | Direct evidence: ↑ probiotics (*Lactobacillus, Bifidobacterium, Pediococcus*); ↓ *Acidaminococcus, Desulfovibrio*; ↑ diversity; normalizes Firmicutes/Bacteroidetesratio | Indirect evidence: No direct lactylation data; SCFAs (butyric/acetic acid) and GPR41/GPR43 activation enhance intestinal barrier and T cell function | Indirect evidence: SCFA production and barrier protection correlate with improved ICI efficacy; probiotic enrichment (Bifidobacterium) supports antitumor immunity | 215. Bai Y, et al. 2023 |
| 36 | OBG; cereal-derived polysaccharide, 155.2 kDa, monosaccharide composition: arabinose/ galactose/ glucose/ xylose/ glucuronic acid | In vivo (BALB/c mice with food allergy-induced colonic injury); FMT validation | Direct evidence: OBG enriches *Lachnospiraceae/Lachnospiraceae_NK4A136_group/Lachnoclostridium*; increases butyrate production; restores gut microbiota diversity | Indirect evidence: No direct lactylation data; butyrate enhances intestinal barrier and regulates Th1/Th2 balance to support immune function | Indirect evidence: SCFA (butyrate) production correlates with improved ICI efficacy; beneficial taxa (Lachnospiraceae) support antitumor immunity | 216. Zhang M, et al. 2024 |
| 37 | *Poria cocos* polysaccharides; fungal polysaccharides, purity 90%, monosaccharide composition: ribose/arabinose/xylose/mannose/glucose/galactose | In vitro (human fecal fermentation) | Direct evidence: ↑ *Lactobacillus/Bifidobacterium/Megamonas/Faecalibacterium*; ↓ *Escherichia-Shigella/Bilophila/Fusobacterium*; ↑ *Firmicutes*, ↓ *Proteobacteria/Desulfobacterota* | Indirect evidence: No direct lactylation data; SCFAs (acetic/propionic acid) and beneficial metabolites (L-cystine) enhance intestinal homeostasis and support immune function | Indirect evidence: SCFA production and microbiota balance correlate with improved ICI efficacy; probiotic enrichment (Bifidobacterium/Lactobacillus) supports antitumor immunity | 217. Zhou X, et al. 2025 |
| 38 | Sulfated polysaccharides from sea cucumber; marine sulfated polysaccharides, main components: fucosylated chondroitin sulfate + fucoidan sulfate | In vivo (humanized microbiota C57BL/6 mice); In vitro (*Lactobacillus* culture) | Direct evidence: Enriches *Lactobacillus*(*L. gasseri, L. reuteri, L. johnsonii*) & unidentified_*Lachnospiraceae*; promotes *Lactobacillus biofilm* & mucus binding; ↑ valeric acid | Indirect evidence: No direct lactylation data; probiotic colonization and metabolite (betaine/hypoxanthine) regulation enhance intestinal homeostasis and immune potential | Indirect evidence: *Lactobacillus* enrichment and metabolic balance correlate with improved ICI efficacy; marine polysaccharide-mediated gut health supports antitumor immunity | 218. Liu Z, et al. 2022 |
| 39 | Representative botanical polysaccharides (SPS2p, GLP, tea polysaccharide, lentinan); multiple botanical sources (*Scutellaria barbata, Ganoderma lucidum*, tea, *Lentinus edodes*) | Cell (CRC cell lines: HT29, SW480, HCT116); Animal (murine models of CRC/colitis); Clinical (cross-study summary) | Direct evidence: Enriches *Bifidobacterium/Lactobacillus/Faecalibacterium*; inhibits pathogenic taxa (*Fusobacterium nucleatum, Escherichia coli*); enhances SCFA production (butyrate/acetate) | Indirect evidence: No direct lactylation data; SCFAs inhibit HDACs (lactylation erasers), thus preserving rather than reducing lactyl marks; outcome is cell-type-dependent. | Direct evidence: Promotes M1 macrophage polarization, activates CD8+ T cells, suppresses Tregs; synergizes with ICIs via NF-κB/PI3K/Akt/mTOR pathway inhibition; enhances antitumor immunity in CRC | 219. Li S, et al. 2024 |
| 40 | *Caulerpa lentillifera* polysaccharide (CLP); marine green algae polysaccharide, 8930.17 kDa, monosaccharide composition: mannose/ galactose/xylose | In vivo (HFD-induced hyperlipidaemic C57BL/6 N mice) | Direct evidence: ↑ SCFA-producers (*Parabacteroides, Ruminococcus_1, Prevotellaceae_UCG-001*); ↓ *Ruminiclostridium, Lachnospiraceae_UCG-006*; ↓ *Firmicutes/Bacteroidetesratio* | Indirect evidence: No direct lactylation data; SCFAs (butyric/acetic acid) and BA metabolism regulation (FXR/TGR5 activation) support metabolic homeostasis and immune potential | Indirect evidence: Gut microbiota-BA axis regulation and lipid metabolism improvement correlate with enhanced antitumor immune microenvironment; SCFA production supports intestinal barrier integrity | 220. Long H, et al. 2025 |
| 41 | Representative polysaccharides (DOP, GP, fucoidan, LBP); multiple sources (plant, fungal, algal) | Cell (RAW 264.7, 4T1, B16F10); Animal (murine models of CRC, melanoma, breast cancer); Clinical (cross-study summary) | Review-summarized evidence: Enriches Bifidobacterium/ Lactobacillus/ Akkermansia muciniphila; inhibits Fusobacterium nucleatum/Escherichia coli; increases SCFA (butyrate/acetate) production # | Indirect evidence: No direct lactylation data; SCFAs inhibit HDACs (lactylation regulators); modulates TME metabolism (glycolysis/lactate) linked to lactylation | Review-summarized evidence: Summarizes reported ICI synergy, CD8+ T cell infiltration, and macrophage repolarization across original preclinical studies | 221. Han S, et al. 2025 |
| 42 | Astragalus polysaccharides (APS); traditional Chinese medicine polysaccharide from *Astragalus membranaceus* | In vivo (melanoma-bearing mice) | Direct evidence: Enriches *Bifidobacterium/Lactobacillus;* reduces pathogenic gut taxa; enhances gut microbiota diversity and SCFA (butyrate) production | Indirect evidence: No direct lactylation data; SCFAs (APS metabolites) inhibit HDACs (lactylation regulators); modulates immune metabolic pathways | Direct evidence: Attenuates immunosuppression; increases CD4+/CD8+ T cell infiltration; reduces Treg cells; enhances antitumor immune response in the tumor microenvironment | 225. Ding G, et al. 2021 |
| 43 | Sea cucumber polysaccharides (SCPs); marine-derived polysaccharides (fucosylated chondroitin sulfate, fucoidan sulfate) from *Stichopus japonicus* | In vivo (MC-38 tumor-bearing C57BL/6 mice); FMT model | Direct evidence: ↑ *Lactobacillus gasseri/L. reuteri/Bifidobacterium pseudolongum*; ↑ diversity (ACE↑, Simpson↓); FMT-validated microbiota-mediated antitumor effect | Indirect: No direct lactylation; ↑ indole-3-carboxylic acid & SCFA-producers; metabolites inhibit HDACs (lactylation regulators) | Direct: Synergizes with anti-PD-1; ↓tumor burden by 30.6% vs anti-PD-1 alone; ↑cytotoxic CD8⁺ T, ↓Tregs; ↑IFN-γ/TNF-α/granzyme B | 226. Li J, et al. 2025e |
| 44 | Selenium-modified polysaccharide (ASPS-e1-Se); from *Acanthopanax senticosus*; MW=1.92×10^4^ Da; GalA/Ara/Gal/Xyl/Rha/Glc/GlcA/Fuc; Se=2.005 mg/g | In vivo (S180 sarcoma‑bearing mice; 100/200/400 mg/kg, 7 days) | Direct evidence: Restores gut microbiota diversity; elevates *Firmicutes/Bacteroidota* ratio; enriches *Lactobacillus*; reduces *Alloprevotella*; promotes propionate/butyrate production | Indirect evidence: No direct lactylation data; SCFA-mediated HDAC inhibition is expected to retain lactyl marks, with effects differing between effector T cells and immunosuppressive populations. | Direct evidence: Inhibits S180 tumor growth; restores thymus/spleen indices; activates NF-*κ*B pathway; enhances TNF- α/IL-6 and reduces IL‑10; boosts antitumor immunity | 227. Chen S, et al. 2026 |
| 45 | Fucoidan (FUC); marine sulfated polysaccharide from *Undaria pinnatifida,* molecular weight: 260 kDa, monosaccharide composition: fucose/galactose/rhamnose | In vivo (Hepa1–6 tumor-bearing C57BL/6 mice); FMT model | Direct evidence: Enriches *Akkermansia/Bifidobacterium/Lactobacillus*; inhibits *Faecalibaculum/Prevotella*; FMT-validated microbiota-dependent antitumor effect | Indirect evidence: No direct lactylation data; SCFAs inhibit HDAC-mediated lactyl mark removal; net effect depends on cell type and tumour compartment. | Direct evidence: Enhances antitumor immunity; increases CD8+ T cells; decreases CD4+ T cells and IDO1 expression; reduces kynurenine/tryptophan ratio | 230. Ren P, et al. 2025a |
| 46 | Ganoderma lucidum/ G. sinense polysaccharides (GPs); β-(1→3)-D-glucan with β-(1→6) branches; 2–4000 kDa | Cell (RAW264.7, MCF-7, CT26); Animal (murine models of colorectal cancer, melanoma, breast cancer); Cross-study | Review-summarized evidence: Summarizes reported gut-microbiota- modulating effects of Ganoderma polysaccharides from primary literature | Indirect evidence: No direct lactylation data; as HDACs erase lactylation, SCFA-driven HDAC inhibition would increase rather than decrease lactyl marks; biological outcome is context-dependent. | Review-summarized evidence: Reviews structure-function relationships of Ganoderma polysaccharides; immunotherapy claims are secondary summaries, not original data | 231. Wu ZW, et al. 2025 |
| 47 | *Gastrodia elata* polysaccharides (GEPs); Mw=292.596 kDa; Glc/Gal/GalA (88.21:4.48:4.40) | In vivo (CTX-induced immunosuppressed BALB/c mice) | Direct evidence: ↑ SCFA-producers (*Muribaculaceae/Prevotellaceae/Bacteroidaceae*); ↓ *Lachnospiraceae/ Ruminococcaceae*; restores *Firmicutes/Bacteroidetesratio*; ↑ SCFAs | Indirect evidence: No direct lactylation data; SCFAs (GEPs metabolites) inhibit HDACs (lactylation regulators); modulates immune metabolic pathways | Direct evidence: Enhances immunomodulatory activity; increases thymus/spleen indices and serum cytokines/immunoglobulins; restores intestinal barrier function (Occludin/ZO-1 upregulation) | 232. Li N, et al. 2023 |
| 48 | *Codonopsis pilosula* alkali-extracted polysaccharide (CPAP); medicinal plant polysaccharide, pH=12 extraction | In vivo (S180 tumor-bearing Kunming mice); multi-omics (16S rDNA, metabolomics, proteomics) | Direct evidence: ↑ *Lactobacillus*(*Lactobacillaceae*); ↓ *Alloprevotella*; maintains diversity; mediates metabolite remodeling (BAs/phospholipids) | Indirect: *Lactobacillus* enrichment implies SCFA production; SCFAs inhibit HDACs (lactylation regulators); modulates purine metabolism | Direct evidence: Inhibits tumor growth (inhibitory rate up to 59.52%); activates T cell immunity; alleviates inflammation via neutrophil degranulation; induces tumor apoptosis through oxidative stress | 233.Fan Y, et al. 2025 |
| 49 | *Polygonatum cyrtonema* Hua polysaccharides (PCPs): Polygonatum cyrtonema‑derived heteropolysaccharide (purity 88.68%, MW 2.95 kDa; composed of Ara, Gal, Glc, Man, Xyl, Rha, GalA, GlcA) | In vivo (chemotherapy‑induced cancer cachexia mice; 32/64 mg/kg, 21 days) | Direct evidence: Restores diversity; ↑ *Firmicutes/Bacteroidota*; ↑ *Lactobacillus, Monoglobus, Ruminococcus, Odoribacter, Enterorhabdus;*↓*Klebsiella, Akkermansia, Enterococcus, Erysipelatoclostridium*; ↑ propionate/butyrate/valerate | Indirect evidence: No direct lactylation data; HDAC inhibition by SCFAs preserves lysine lactylation; functional outcome varies by cell subset (effector T cells vs tumour cells/Tregs/MDSCs). | Direct evidence: Alleviates muscle atrophy and fat lipolysis; restores mitochondrial function; inhibits atrogin‑1/MuRF1; enhances intestinal tight junction proteins (claudin-1, occludin, ZO - 1); suppresses LPS/TNF-α/NF-κB/IL-6 | 234. Zhou R, et al. 2025 |
| 50 | *Moringa oleifera* leaf polysaccharides (MOLP): from Moringa oleifera leaves, composed of galactose, glucose, arabinose; Mw=17.35 kDa | In vitro (tumor cells: LLC; immune cells: RAW 264.7, BMDMs, T cells); in vivo (LLC tumor-bearing C57BL/6 mice) | Indirect evidence: Improves immune homeostasis, potentially regulating gut microbiota diversity | Indirect evidence: Modulates macrophage metabolism and inflammatory responses in TIME, potentially influencing lactylation (no direct modification data) | Direct: Targets TLR4 → M2→M1 TAMs; ↑CD4⁺/CD8⁺ T infiltration & cytotoxicity; inhibits angiogenesis; enhances lung cancer antitumor immunity | 241. Wang S, et al. 2023 |
| 51 | *Spirulina subsalsa* polysaccharides (SPS) hydrogel: Self-assembling heteropolysaccharide (nanoporous structure, Mw>20,000 kDa) | In vitro (immune cells: RAW 264.7 macrophages) | Indirect evidence: Microalgal polysaccharide with prebiotic potential, potentially regulating gut microbiota diversity | Indirect evidence: Modulates macrophage metabolism and inflammatory responses, potentially influencing lactylation (no direct modification data) | Direct evidence: Balances TNF-α/IL-10 secretion; enhances macrophage phagocytosis and NO production; nanoporous structure supports drug delivery for combined immunotherapy | 242. Zhao Y, et al. 2025 |
| 52 | Chinese yam polysaccharide (CYP): bioactive plant polysaccharide extracted from *Dioscoreae Rhizoma* (Chinese yam) | Cell: RAW264.7; Animal: MC38/CT26 (C57BL/6 & BALB/c); Preclinical | Direct evidence: Oral CYP remodels flora; ↑ *Clostridia_UCG-014, Actinobacteria*; ↓ *Enterorhabdus*, *Desulfovibrionaceae*; antibiotic ablation eliminates CYP's immunopotentiating effects | Indirect: No direct lactylation; reshapes TME via purine/Trp/linoleic acid microbial metabolites | Direct: Synergizes with anti-PD-1; suppresses CRC growth & prolongs survival; M2→M1; ↑intratumoral CD8^+^ T; ↓deoxyguanosine (pro-tumor metabolite) | 243. Zhang Y, et al., 2025 |
| 53 | Astragalus polysaccharide (APS)-gold NPs (APS-AuNP); Lentinan (LNT)-ursolic acid (UA) NPs (LNT-UA); *Ganoderma lucidum* polysaccharide (GLP)-gold NPs (GLP-Au) | In vitro (DCs); in vivo (4T1 tumor-bearing mice); in vivo (CT26 tumor-bearing mice); in vivo (4T1 tumor-bearing mice) | Indirect evidence: APS modulates gut microbiota diversity, potentially enhancing beneficial taxa; LNT as prebiotic regulates gut microbiota composition; GLP enhances gut microbiota balance | Indirect: Modulates DC/T-cell & macrophage metabolism; potentially influencing lactylation (no direct data) | Direct: Promotes DC maturation & CD4⁺/CD8⁺ T; induces ICD; M2→M1; synergizes with α-CD47; reverses chemo-induced immunosuppression | 244. Han MM, et al. 2024 |
| 54 | Glycyrrhiza polysaccharides (GCP): Heteropolysaccharide (*Glycyrrhiza uralensis*, Mw=5.5×10⁴ Da) | In vitro (CT-26 colon cancer cells); in vivo (CT-26 tumor-bearing BALB/c mice) | Direct evidence: Upregulates *Firmi- cutes/Proteobacteria*, downregulates *Bacteroidetes/ Tenericutes*; increases *Akkermansia, Parabacteroides*; restores gut microbiota diversity | Indirect evidence: Regulates γδT cell metabolism and cytokine secretion via TLRs/NF-κB pathway, potentially influencing lactylation (no direct modification data) | Direct evidence: Promotes γδT cell activation (NKG2D^+^/CD27^+^) and IFN- γ secretion; enhances CD3^+^ T cell infiltration; activates TLR2/ TLR4/ TLR6 and NF-κB pathway | 245. Chen Y, et al. 2024 |
| 55 | Astragalus polysaccharide (ASP)-based pH/ROS dual-responsive nanogels (BAI@ASPOBA); ASP (Mw=66 kDa/2922 Da) + OBA cross-linker + baicalein (BAI) | In vitro (tumor cells: A549, HepG2; immune cells: RAW264.7 macrophages); in vivo (A549 tumor-bearing zebrafish) | Indirect evidence: ASP modulates gut microbiota diversity, potentially enhancing beneficial taxa | Indirect evidence: Regulates macrophage/T-cell metabolism and ROS production, potentially influencing lactylation (no direct modification data) | Direct: Synergistic chemo-immunotherapy; BAI induces apoptosis; ASP activates macrophages (↑co-stimulatory molecules, ↑NO); inhibits A549 growth (IR=79.54%) in zebrafish | 248. Wang S, et al. 2025 |

Note: Abbreviations: ICI, immune checkpoint inhibitor; TME, tumor microenvironment; SCFA, short‑chain fatty acid; MDSC, myeloid-derived suppressor cell; Treg, regulatory T cell; DC, dendritic cell; NK, natural killer cell; FMT, fecal microbiota transplantation; CRC, colorectal cancer; NSCLC, non-small cell lung cancer; HCC, hepatocellular carcinoma.

‑ Direct evidence: Original primary experimental data from in vitro, in vivo animal or human patient‑cohort studies.

‑ Indirect evidence: Correlative observation, bioinformatic analysis, or mechanistic link without direct molecular detection of lactylation or direct microbiota perturbation experiments.

‑ Review‑summarized evidence: Secondary summary derived from review articles, not primary experimental results.

\# Among these polysaccharides, fucoidan has primary in-vivo tumor-bearing-mouse evidence demonstrating gut‑microbiota-dependent anti-PD-1 immunotherapy efficacy (Li H et al., *Food & Function*, 2024, DOI: 10.1039/D3FO04807A). Data for DOP, GP and LBP are aggregated from scattered non-tumor or partial-model studies and do not fully cover all tumor types listed in this table row.
